# Immune checkpoint-based biomarkers for therapeutic response in patients with multiple sclerosis

**DOI:** 10.3389/fimmu.2025.1694021

**Published:** 2025-10-20

**Authors:** MariPaz López-Molina, Gabriel Torres Iglesias, Gonzalo Sáenz de Santa María-Diez, Jaime Valentín-Quiroga, Fernando Laso-García, Rebeca Gallego, Javier Pozo-Novoa, Beatriz Chamorro, Eduardo López-Collazo, Inmaculada Puertas, Exuperio Díez-Tejedor, María Gutiérrez-Fernández, Laura Otero-Ortega

**Affiliations:** ^1^ Neurological Sciences and Cerebrovascular Research Laboratory, Department of Neurology, Neurology and Cerebrovascular Disease Group, Neuroscience Area of Hospital La Paz Institute for Health Research IdiPAZ, La Paz University Hospital, Universidad Autónoma de Madrid, Madrid, Spain; ^2^ Immune innate response group, La Paz Hospital Institute for Health Research IdiPAZ, La Paz University Hospital, Madrid, Spain; ^3^ Tumor Immunology Lab, IdiPAZ, La Paz University Hospital, Madrid, Spain; ^4^ Centro de Investigación Biomédica en Red (CIBER), Respiratory Diseases (CIBRES), Madrid, Spain; ^5^ UNIE Universidad, Arapiles 4, Madrid, Spain

**Keywords:** immune checkpoint molecules, immune cell subpopulations, multiple sclerosis, therapeutic response, biomarkers

## Abstract

**Introduction:**

Although numerous disease-modifying treatments have been introduced for multiple sclerosis (MS), approximately 25% of patients experience therapeutic failure. This underscores the urgent need for reliable, minimally invasive biomarkers to predict treatment response at early stages. This study aimed to investigate 22 circulating immune cell subpopulations and their immune checkpoint (IC) expression profiles to identify early immunological biomarkers indicative of therapeutic failure in MS patients.

**Methods:**

In this observational and prospective study, 119 patients with relapsing-remitting MS were enrolled, including 69 responders and 50 non-responders, and 29 healthy controls. Spectral flow cytometry was used to immunophenotype 22 immune cell subpopulations and quantify the expression of co-stimulatory and co-inhibitory ICs before and at three months post-treatment initiation. Their correlation with therapeutic response over 12 months in MS patients was also analyzed. The response to treatment was evaluated using the No Evidence of Disease Activity composite, which includes clinical relapses, new lesions on MRI and progression of motor disability.

**Results:**

We identified differential IC expression patterns between MS patients and healthy controls, revealing specific ICs involved in the disease. Within the MS cohort, we observed differences between treatment responders and non-responders. Responders exhibited higher CD70 expression on Natural Killer^bright^ cells. Additionally, elevated inhibitory CTLA-4 levels on CD20^-^CD27^+^ B cells may serve as biomarker for disability progression. BTLA expression on CD20^+^CD27^-^ B cells was associated with relapse events, and PD-L1 expression on Natural Killer^bright^ cells appeared to be a potential biomarker for progression independent of relapse activity (PIRA).

**Discussion:**

These findings highlight that specific immune cell subpopulations and their IC expression profiles can serve as valuable, early, and minimally invasive immunological markers for predicting therapeutic response in MS patients.

## Graphical Abstract

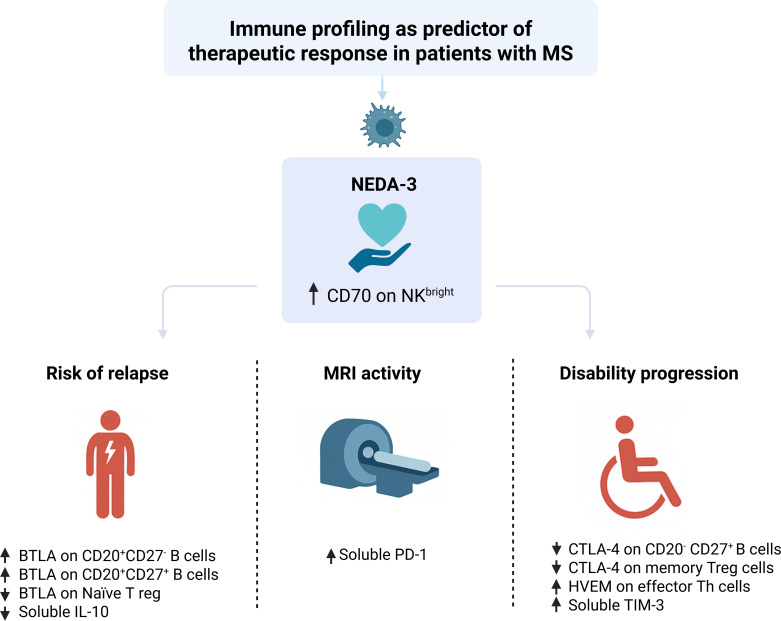

## 1 Introduction

Multiple sclerosis (MS) is an immune-mediated neurological disease of the central nervous system (CNS) that affects 2.8 million people worldwide (1). Although the etiology is unknown, the most accepted hypothesis is that MS is a multifactorial entity that occurs when certain environmental factors act on genetically predisposed individuals. This interplay between genetic predisposition and environmental exposure results in immune system dysfunction that initiates with a loss of immune tolerance to CNS myelin antigens. T cells mistakenly recognise CNS myelin antigens as “foreign” (2) probably due to a defect in the recognition of antigens presented by antigen-presenting cells to T cells via the immunological synapse (3). When the synapse becomes overly sensitive, it can result in the inappropriate activation of autoreactive T and B cells, which then target the body’s own CNS myelin (4). The immunological synapse functions by integrating activating and inhibitory signals, which are known as immune checkpoint (IC) molecules. Dysregulation of these signals, such as the overactivation of costimulatory pathways or under activation of inhibitory checkpoints, can lead to a breakdown in self-tolerance toward myelin (5). When this occurs, autoreactive T cells, together with B cells and monocytes, cross the blood-brain barrier and infiltrate the brain and spinal cord tissue. Once inside the CNS, they release cytokines and autoantibodies, which activate astrocytes and microglia, degrade the extracellular matrix and induce apoptosis in oligodendrocytes. This inflammatory immune response leads to demyelination in the CNS, resulting in axonal and neuronal loss (6).

These lesions in CNS are associated with varying levels of disability, making patients highly vulnerable and dependent. It is essential to provide these individuals with the most effective treatments to reduce disease activity and disability, improving their quality of life for them and their families. Over the past 20 years, researchers have developed 20 different disease modifying therapies (DMTs) to help treat MS by controlling the immune system’s aggressive response against the CNS (7). However, despite these therapeutic advancements, more than 25% of patients do not respond adequately to the initial treatment and must switch to a different drug, which can be an uncertain and challenging process. Although serum neurofilament light chains are a promising biomarker of neuroinflammation and treatment response, this study was conducted to provide additional robustness to current biomarkers. Currently, there are no blood biomarkers implemented in clinical practice to help neurologists select the most effective treatment for each patient or to detect therapeutic failure early enough to allow timely adjustments (8). Instead, treatment failure is monitored by waiting for new relapses or detecting new damage in the CNS, which puts at risk the patient’s health and integrity.

DMTs have been shown to influence the expression of IC molecules in patients with MS, highlighting their potential as biomarkers of disease activity and treatment response. Interferon-β treatment has been associated with upregulation of CD40, CD86, and PD-L1 ligands in relation to clinical response to treatment (9) and with reduced serum levels of CD40L correlating with response to treatment (10). Dimethyl fumarate has been reported to downregulate CD40 on dendritic cells and increase CD27 expression in B cells (11). Additionally, altered PD-1/PD-L1 expression profiles correlate with disability progression and increased PD-1^+^ T follicular helper cell frequencies in cerebrospinal fluid have been linked to worsening disease (12–15). Similarly, functional alterations in TIM-3 and CTLA-4 have been associated with MS progression and therapeutic response (16–20). Moreover, changes in soluble CD40L levels following treatment with natalizumab, glatiramer acetate, or interferon-β suggest its utility as a marker of therapeutic efficacy (21–23). Collectively, these findings underscore the potential of ICs as biomarkers for MS diagnosis, disease staging, prognosis, and monitoring of therapeutic responses, thereby paving the way toward personalized strategies in managing this complex autoimmune disorder.

This study aims to establish a novel strategy for the early assessment of treatment response in patients with MS, enabling clinicians to anticipate clinical events that could lead to irreversible disability. To achieve this, we sought to identify early biomarkers that reflect how treatment is influencing the patient’s immune system and whether these immunological changes are associated with subsequent treatment response or failure. We propose a detailed analysis of 22 immune subpopulation proportions and their expression of activating and inhibitory ICs. By analysing the expression patterns of these checkpoints in specific immune subpopulations, we can assess how treatments are modulating the immune system. This information can help identify early signs of therapeutic success or failure, enabling timely intervention to optimise patient outcomes.

## 2 Material and methods

### 2.1 Study design

This was an observational, longitudinal, prospective clinical study involving patients with a confirmed diagnosis of relapsing-remitting MS based on the McDonald criteria (24), conducted between May 2021 and October 2024. Each patient attended three visits at specific time points for correlative assessments: pre-treatment (prior to initiating treatment) and post-treatment (at 3 and 12 months after treatment onset). These visits were aligned with routine clinical practice and did not cause additional discomfort for participants. All clinical assessments and outcome measurements were conducted exclusively by experienced neurologists. Participants were selected according to the following criteria:

Inclusion criteria: male and female patients over 18 years old with RRMS initiating or changing their DMT (Interferons, Teriflunomide, Dimethyl fumarate (DMF), Cladribine (CdA), Natalizumab (NTZ) and anti-CD20 treatments).

Exclusion criteria: patients with progressive MS, current substance dependence, severe comorbid conditions, with other immune-mediated disease, pregnant or breastfeeding women, and those participating in pharmacological treatment trials or receiving chemotherapy.

As a control group, the study included healthy controls (HCs) who voluntarily agreed to participate.

*A priori* sample size calculation using G*Power 3.1 indicated that a minimum of 114 patients was required to achieve 80% power at a 0.05 significance level. The present study included 119 patients.

### 2.2 Ethics approval

This study was approved by the Research Ethics Committee of La Paz University Hospital (PI-4675). Data management was conducted in compliance with Spanish Law 14/2007 of July 3rd on Biomedical Research, ensuring the confidentiality of all personal information. The study adhered to the ethical principles outlined in the World Medical Association’s Declaration of Helsinki. Informed consent was obtained from all participants.

### 2.3 Demographic and clinical data

The following demographic and clinical information was collected from participants: sex; age; disease duration; baseline and 12 months after treatment scores on the Expanded Disability Status Scale (EDSS); Nine-Hole Peg Test (9-HPT) and current treatments.

### 2.4 Clinical variables

The potential relationship between the proportion of cellular subpopulations and their expression of ICs before and after treatment initiation with new relapses, new lesions on magnetic resonance image (MRI), and EDSS and 9HPT progression occurred throughout the 12-month follow-up period of the study was analyzed. In addition, progression independent of relapse activity (PIRA) and relapse-associated worsening (RAW) were also examined.

Moreover, treatment response was evaluated at the 12-month follow-up based on the ECTRIMS guidelines (25) and the NEDA-3 (No Evidence of Disease Activity-3) criteria. Patients were classified as “responders” if they had no relapses, no MRI activity, and no disability progression (assessed using the EDSS and the 9-HPT) during 12 months of follow-up (26). Those presenting any of these three signs of EDA (Evidence of Disease Activity) for 12 months were categorized as “non-responders”. Lastly, this study also explores PIRA defined by confirmed disability accumulation on the EDSS scale at 6 months during a relapse-free period; and relapse-associated worsening (RAW).

### 2.5 Sample collection

A total of 10 mL of blood was collected from patients with RRMS, distributed into 2 EDTA tubes. For those who had received previous DMTs, the required washout period for each treatment was observed according to standard clinical practice. Peripheral blood was immediately centrifuged at 3000 g for 15 minutes at 4 °C. Plasma was collected in tubes and stored at temperature of -80 °C until further analysis. Peripheral blood mononuclear cells (PBMCs) were isolated using a density gradient separation method with Ficoll, then collected in 2 cryotubes and stored at N_2_ until further analysis.

### 2.6 Immunophenotypic profiling of immune cell subpopulations

PBMCs were thawed, washed, and directly processed for staining. Cell viability was determined using a Live and Dead viability dye, and only samples with ≥80% viability was included in the analysis. To identify the specific subpopulations that influence treatment response mechanisms, the immune subsets proportion was characterised and compared across different groups. For this purpose, the following 22 subpopulations were studied according to methods previously described (27–29) in the literature: classic monocytes CD14^hi^CD16^-^, non-classic monocytes CD14^lo^CD16^+^, intermediate monocytes CD14^lo-hi^CD16^+^, inflammatory dendritic cells (InfDCs) CD14^+^HLADR^+^CD1C^+^, CD19^+^CD20^+^CD27^-^ B cells, CD19^+^CD20^+^CD27^+^ B cells, CD19^+^CD20^-^CD27^+^ B cells, memory Treg cells CD3^+^CD4^+^CD25^+^CD127^-^CD27^+^CD45RA^-^, naïve Treg cells CD3^+^CD4^+^CD25^+^CD127^-^CD27^+^CD45RA^+^, naïve Th cells CD4^+^CD3^+^CD4^+^CD27^+^CD45RA^+^, memory Th cells CD4^+^CD3^+^CD27^+^CD45RA^-^, effector Th cells CD3^+^CD4^+^CD27^-^CD45RA^-^CD27^-^, naïve CD8^+^ T cells CD3^+^CD8^+^CD45RA^+^CD27^+^, memory CD8^+^ T cells CD3^+^CD8^+^CD45RA^-^CD27^+^, effector CD8^+^ T cells CD3^+^CD8^+^CD45RA^-^CD27^-^, terminal effector CD8^+^ T cells CD3^+^CD8^+^CD45RA^+^CD27^-^, CD56^+^CD3^+^ cells, NK CD16^-^CD56^bright^, NK CD16^dim^CD56^bright^, NK CD16^bright^CD56^dim^, NK CD16^bright^CD56^-^, NK CD16^dim^CD56^dim^, NK CD16^-^CD56^dim^ using the Cytek™ Aurora flow cytometer. The data obtained were processed using FlowJo™ v_10.10.0 software (FlowJo, LLC, USA), which allows for the processing, analysis and visualisation of flow cytometry data. For specific information regarding the fluorochromes used in the designed panel, please refer to **Supplementary Table 1**.

### 2.7 Analysis of surface immune checkpoint expression

The expression of the following co-stimulatory and co-inhibitory ICs was analyzed in each cell subset and compared between groups: CD80-CD28/CTLA-4, CD70-CD27, PD-1-PD-L1, HVEM-BTLA-4, CD40 and OX40. This expression was analyzed using the Cytek™ Aurora flow cytometer (Cytek). The data obtained were processed using FlowJo™ v_10.10.0 software (FlowJo, LLC, USA). For specific information regarding mean fluorescence intensity (MFI) and event counts please refer to **Supplementary Tables 2** and **3**.

### 2.8 Study of soluble immune checkpoint levels

The plasma levels of the following soluble co-stimulatory and co-inhibitory molecules sCD25 (IL-2Ra), 4-1BB, CD86 (B7.2), TGF-β1, CTLA-4, PD-L1, PD-1, Tim-3, LAG-3, Galectin-9 were analyzed using the LEGENDplex™ HU Immune Checkpoint Panel 1 - S/P (Biolegend, USA) w/VbP on the BD FACS Calibur™ flow cytometer.

### 2.9 Study of soluble cytokines levels

The plasma levels of the following soluble cytokines IL-5, IL-13, IL-2, IL-6, IL-9, IL-10, IFN-y, TNF-α, IL-17A, IL-17F, IL-4, IL-22 were analyzed using LEGENDplex™ HU Th Cytokine Panel (Biolegend, USA) w/VbP V02 on the BD FACS Calibur™ flow cytometer.

### 2.10 Statistical analysis

Quantitative results are expressed as mean ± standard deviation (SD). Prior to analysis, all variables were categorized as either numerical or categorical. Statistical analyses were conducted using R version 4.5.0 (R Core Team, Austria), RStudio 2025.05.0 (Posit PBC, USA), and SPSS version 26 (IBM Corp., USA).

Normal distribution of continuous data was tested using either the Shapiro-Wilk test or the Kolmogorov–Smirnov test with Lilliefors correction, depending on sample size. Variables failing to meet normality criteria were treated as non-parametric. For comparisons between two groups, either the unpaired Student’s t-test or the Mann-Whitney U test was applied based on the distribution of the data. Categorical data were analyzed using Chi-square or Fisher’s exact test, depending on expected frequencies. All *p*-values were adjusted using the Benjamini–Hochberg method to control the false discovery rate. When comparing more than two groups, one-way ANOVA or the Kruskal-Wallis test was employed, followed by appropriate *post hoc* tests (Tukey’s HSD or Dunn’s test). Correlations between variables were assessed using Pearson’s or Spearman’s correlation coefficients, depending on the normality of the data.

To assess the discriminatory capacity of individual immune parameters, Receiver Operating Characteristic (ROC) curve analyses were performed. The area under the curve (AUC) was calculated as a measure of diagnostic accuracy. Optimal cut-off points were determined using the Youden index, and corresponding sensitivity and specificity values were reported.

To explore the association between specific clinical outcomes and immune parameters, linear regression models were applied. The dependent variable in each model was the immune marker level measured three months after treatment initiation, and the independent variable was the presence or absence of a clinical event. Analyses were stratified by treatment response status (responders vs. non-responders) and were specifically focused on the non-responder group to identify immune correlates of differential clinical manifestations (relapse, new MRI lesion, or disability progression). Formula: biomarker~relapses+EDSS+lesions.

Regression outputs included the estimated coefficient (estimate), standard error, 95% confidence interval (CI), and associated *p*-value. A *p*-value < 0.05 was considered statistically significant. All figures and plots were created using GraphPad Prism 8 (GraphPad Software, USA) , RStudio 2025.05.0 (Posit PBC, USA) and BioRender.com. Statistical significance was denoted as: *p* < 0.05 (*), p < 0.01 (**), and p < 0.001 (***).

## 3 Results

**Figure 1:**
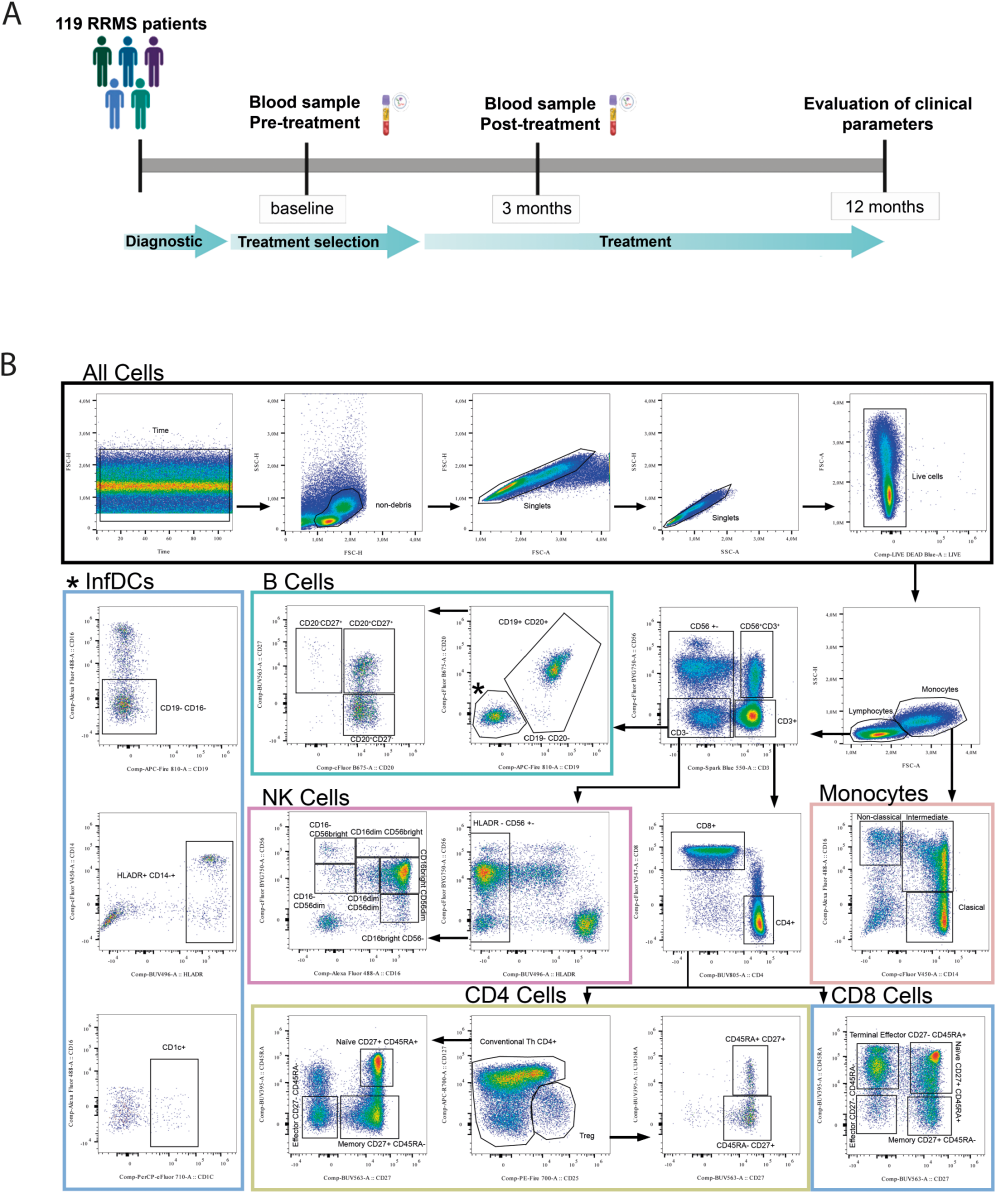
**(A)** Schematic representation of the study design in 119 patients with RRMS. Blood samples were collected at baseline (pre-treatment) and at 3 months (post-treatment), while clinical parameters of treatment response and disease progression were evaluated at 12 months after treatment initiation. **(B)** Gating strategy for spectral cytometry. The gating strategy used to identify the 22 immune cellular subsets is represented. All data presented is derived from frozen peripheral blood mononuclear cells of one HC individual of the study.

The present study included 119 patients with RRMS who started new DMT (**Figure 1A**), 69 patients were considered as responders, 50 patients as non-responders and there were 29 healthy controls. Patient characteristics are summarised in **Table 1**.

**Table 1:** Demographic and clinical data of the study participants.

	RRMS patients (n=119)	HCs (n=29)	*p*-value
Demographics			
Women. n (%)	71 (59.7%)	14 (48.3%)	0.299
Age. years	42.82 (10.08)	32.2 (10.29)	**0.001**

	Responders (n=69)	Non-responders (n=50)	*p*-value
Demographics			
Women. n (%)	37 (53.6%)	34 (68%)	0.115
Age. years	43.16 (10.06)	42.70 (10.19)	0.863
Clinical data			
Time from diagnosis. months	144.87 (130.23)	144.35 (115.24)	0.770
Baseline EDSS	1.57 (1.68)	2.14 (2.19)	0.215
Relapses, 12 mo.	–	0.30 (0.63)	–
New lesions, 12 mo.	–	1 (1.34)	–
Baseline 9HPT_dom. sec	24.46 (8.29)	28.37 (27.43)	0.353
Treatments received			
Natalizumab. n (%)	15 (21.7%)	10 (20%)	
Teriflunomide. n (%)	4 (5.8%)	4 (8%)	
Interferon. n (%)	7 (10.1%)	1 (2%)	
S1P modulators (%)	2 (2.9%)	1 (2%)	
Dimethyl fumarate. n (%)	12 (17.4%)	8 (16%)	
Anti-CD20. n (%)	13 (18.8%)	10 (20%)	
Cladribine. n (%)	16 (23.2%)	16 (32%)	
Previously treatments received			
Natalizumab. n (%)	3 (7.7%)	5 (16.1%)	
Teriflunomide. n (%)	7 (17.9%)	3 (9.7%)	
Interferon. n (%)	14 (35.9%)	4 (12.9%)	
S1P modulators (%)	–	–	
Dimethyl fumarate. n (%)	9 (23.1%)	10 (32.3)	
Anti-CD20. n (%)	–	2 (6.5%)	
Cladribine. n (%)	1 (2.6%)	–	
Alemtuzumab. n (%)	1 (2.6%)	1 (3.2%)	
Glatiramer Acetate. n (%)	4 (10.3%)	6 (19.4%)	

We compared the distribution of patients with responder and non-responder status across different initial treatments. No statistically significant association was found between treatment group and responder/non-responder status (Chi-square test, χ^2^ (6) = 4.10, *p* = 0.664). We compared the distribution of patients with responder and non-responder status across different previously treatments. No statistically significant association was found between treatment group and responder/non-responder status (Chi-square test, χ^2^ (7) = 10.3, *p* = 0.171).

All values are mean (SD) unless otherwise noted. mo.=months. dom.=dominant hand.

Mann–Whitney U test for continuous variables and Fisher’s exact test for categorical variables were employed to determine statistically significant differences between groups. N, number; SD, standard deviation; EDSS, expanded disability status scale.

Bold means *p* < 0.05.

### 3.1 Phenotypic characterisation of immune populations

The following steps were taken to design a spectral cytometry panel capable of detecting all the essential markers for identifying immune system cells most relevant to the pathogenesis of MS. Initially, data cleaning was performed to ensure the accuracy of the analysis. A time graph was generated to monitor the number of events over time, ensuring stable data acquisition. Periods of instability or interruptions were identified, and a gate was drawn around the events acquired during stable periods, excluding those from perturbation periods. Debris was identified using forward scatter and side scatter plots, where debris events, due to their smaller size and complexity, were excluded from further analysis. Non-debris events were selected for subsequent gating. To ensure the purity of the single-cell population, doublets were excluded using FSC-H vs. FSC-A and SSC-H vs. SSC-A plots. Single cells were identified as a well-defined population along a diagonal line, and a gate was drawn around this population to exclude aggregates. Live cells were identified using a live/dead marker. Following the selection of live cells, the gating strategy was applied to differentiate between monocytes and lymphocytes. Monocytes were differentiated into three subsets based on CD14 and CD16 expression: CD14^hi^CD16^-^ classical monocytes, CD14^lo-hi^CD16^+^ intermediate monocytes, and CD14^lo^CD16^+^ non-classical monocytes. Lymphocytes were gated and further subpopulations were determined using CD3 and CD56 markers. T cells were identified by CD3^+^ expression. CD4^+^ T cells were further analyzed for CD25^+^ expression to distinguish T helper (Th) and regulatory T CD25^+^ (Treg) cells. Th cells were categorised as: CD27^+^CD45RA^+^ naïve Th, CD27^-^CD45RA^-^ effector Th, memory Th CD27^+^CD45RA^-^ and Treg cells were classified as: CD27^+^CD45RA^+^ naïve Treg, CD27^+^ CD45RA^-^ memory Treg. CD8^+^ T cells were differentiated as: CD27^+^CD45RA^+^ naïve cytotoxic T cells, CD27^-^CD45RA^-^ effector cytotoxic T cells, CD27^-^CD45RA^+^ terminal effector cytotoxic T cells, CD27^+^CD45RA^-^memory cytotoxic T cells. CD3^-^ cells were analyzed using CD19^+^ and CD20^+^ markers to identify B cells. B cells were further differentiated into: CD20^-^CD27^+^ B cells, CD20^+^CD27^-^ B cells, CD20^+^CD27^+^ B cells. InfDCs were identified by gating CD20^-^CD19^-^ cells, confirmed with CD16^-^ markers, and characterised by HLA-DR^+^CD14^+-^ and CD1c^+^ expression. Natural Killer (NK) cells were identified by CD56^+^ expression and confirmed using HLA-DR^-^. Six NK subpopulations were identified based on CD16 and CD56 expression: CD16^-^CD56^bright^, CD16^dim^CD56^bright^ collectively categorized as NK^bright^, CD16^bright^CD56^dim^, CD16^bright^CD56^-^, CD16^dim^CD56^dim^, CD16^-^CD56^dim^ collectively categorized as NK^dim^ (**Figure 1B**). Once these cells were identified, we analyzed the expression of the following immune ICs by adding the corresponding markers to this panel, activators: CD80, CD28, CD70, CD27, CD40, OX40, HVEM and inhibitors: PD-1, PD-L1, HVEM, BTLA, and CTLA-4.

### 3.2 Cell population proportions and their IC expression differ in MS patients and HCs

**Figure 2:**
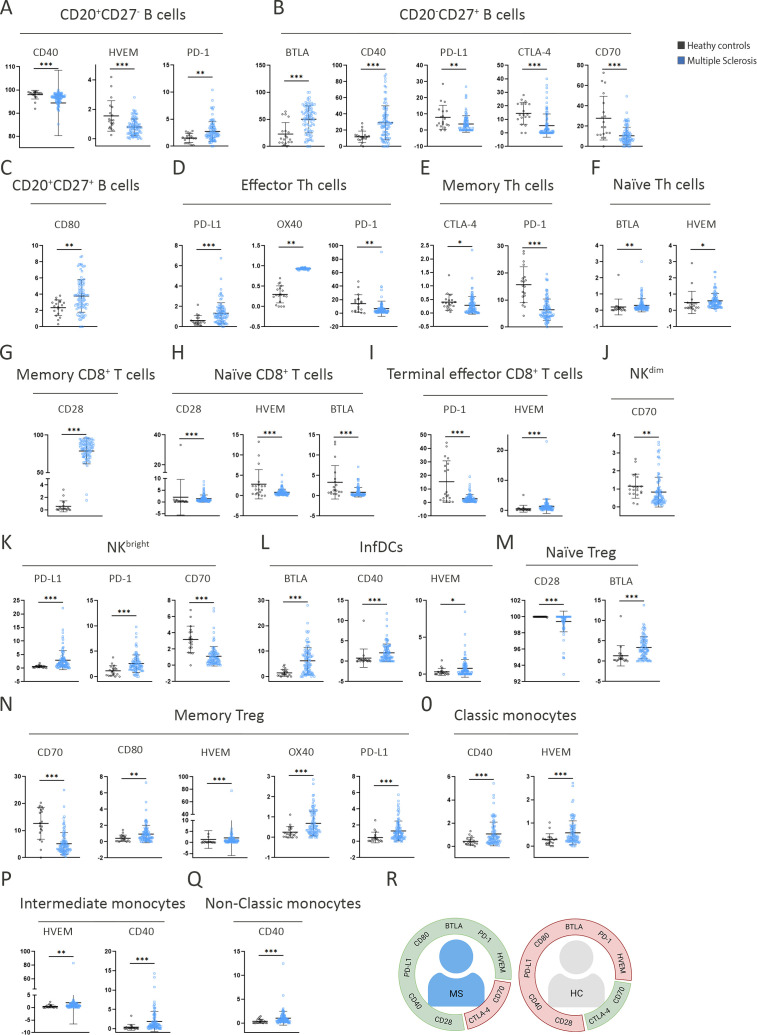
The pattern of activation and inhibition of immune system cells of RRMS patients shows differences with the pattern shown by HCs. IC expression between HCs and patients with MS across subcellular subsets. **(A–C)** B cells; **(D–F)** T helper cells; **(G–I)** CD8^+^ T cells; **(J, K)** Natural killer; **(L)** InfDCs; **(M, N)** Treg cells; **(O–Q)** Monocytes. Data are mean ± SD. *p < 0.05. **p < 0.01. ***p < 0.001. **(R)** Schematic summary of overexpressed (in green) and underexpressed (in red) ICs.

Cell population proportions and IC expression differed significantly in untreated patients with MS compared to HCs (**Figures 2A–Q**, **Supplementary Table S4**). In CD20^+^CD27^-^ B cells, MS patients showed lower CD40 and HVEM expression and higher PD-1 expression than HCs (**Figure 2A**). In CD20^+^CD27^+^ B cells, CD80 expression was higher in MS patients compared to HCs (**Figure 2C**).

The most pronounced differences were observed in CD20^-^CD27^+^ B cells, where BTLA and CD40 expression were higher in MS patients, while PD-L1, CTLA-4, and CD70 were significantly reduced compared to HCs (**Figure 2B**). In effector Th cells, PD-1 expression was lower in MS patients relative to HCs (**Figure 2D**), and in memory Th cells, both CTLA-4 and PD-1 were downregulated in MS patients (**Figure 2E**).

In naïve Th cells, BTLA and HVEM expression were elevated in MS patients versus HCs (**Figure 2F**). Terminal effector CD8^+^ T cells from MS patients exhibited reduced PD-1 expression, while HVEM expression remained slightly increased compared to controls (**Figure 2I**). In memory CD8^+^ T cells, CD28 expression was increased in MS, whereas naïve CD8^+^ T cells showed reduced CD28, BTLA, and HVEM expression compared to HCs (**Figures 2G, H**).

In the myeloid lineage, InfDCs from MS patients displayed higher CD40 and HVEM expression relative to HCs (**Figure 2L**). Classical, intermediate, and non-classical monocytes all showed increased CD40 expression in MS, with higher HVEM expression in classical and intermediate subsets as well (**Figures 2O–Q**).

In NK cells, both NK^bright^ and NK^dim^ subsets revealed altered IC profiles in MS. NK^bright^ cells exhibited increased PD-L1 and PD-1, while CD70 expression was reduced compared to HCs. Similarly, CD70 expression in NK^dim^ cells was also lower in MS patients (**Figures 2J, K**).

Finally, in the Treg compartment, memory Tregs in MS patients showed higher PD-L1 expression, whereas naïve Tregs exhibited comparable CD28 expression but increased BTLA expression relative to HCs (**Figures 2M, N**). **Figure 2R** summarizes the most prominently upregulated and downregulated activating and inhibitory markers in MS patients compared to healthy controls.

### 3.3 Effects of DMTs on IC expression and immune cell subsets

**Figure 3:**
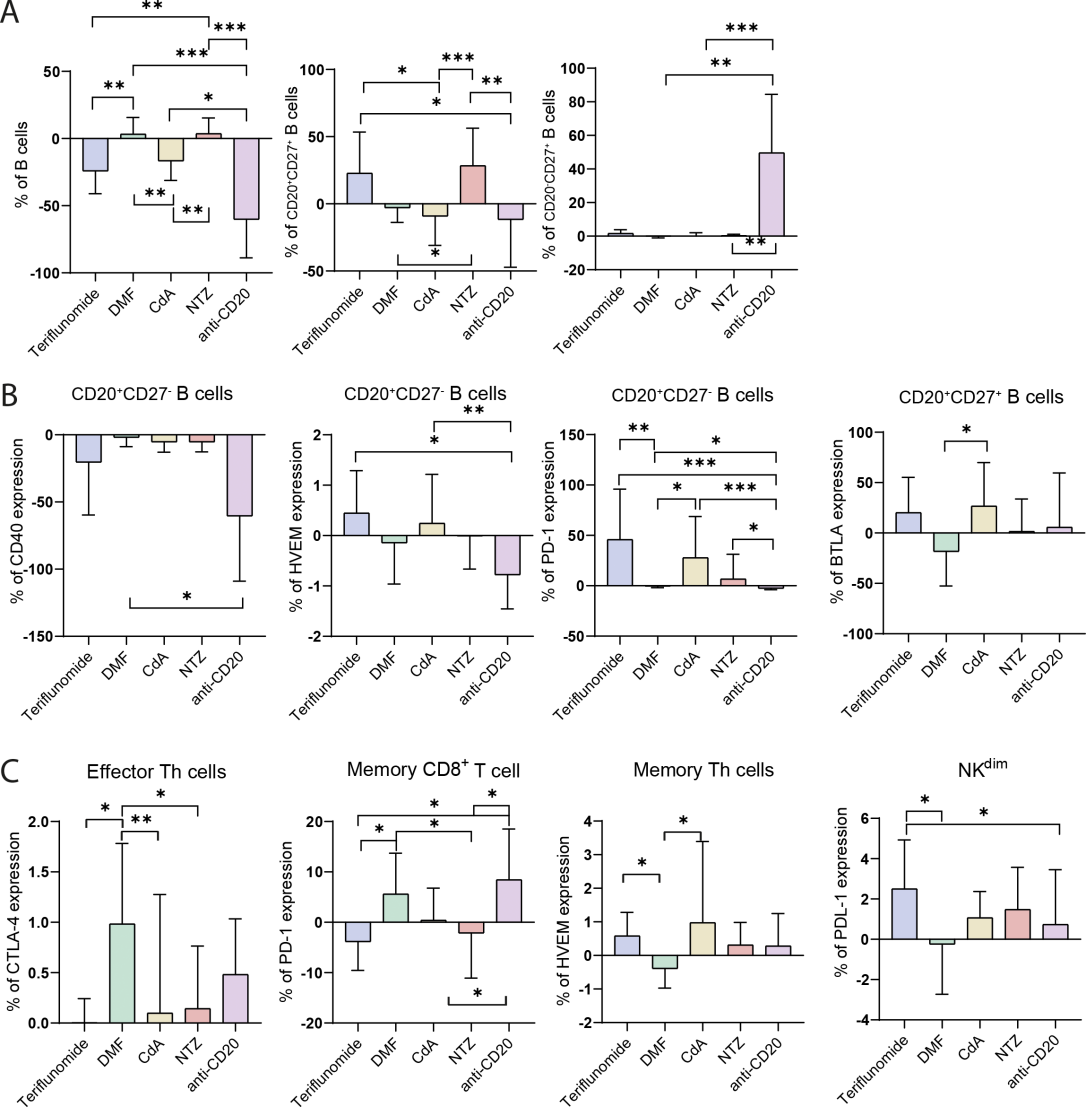
Effects of DMTs on cell percentages and IC expression from baseline to three months after treatment initiation. **(A)** B cells populations; **(B)** ICs expression in B cells populations; **(C)** ICs expression in Th, CD8^+^ T and NK cells populations. Data are presented as % Mean ± SD. Color coding: NTZ (red bars), teriflunomide (blue bars), DMF (green bars), anti-CD20 antibody (pink bars) and CdA (yellow bars). Data are mean ± SD. *p < 0.05. **p < 0.01. ***p < 0.001.

Both ICs expression and cell populations were analyzed at baseline (pre-treatment) and three months post-treatment. Through these, the phenotypic changes in immune system cells due to the DMTs [NTZ, teriflunomide, DMF, anti-CD20 antibodies, and CdA] were analyzed (**Figure 3A**, **Supplementary Table S5**).

Notably, the most pronounced differences between treatments were observed in B cell percentages. Total B cell decreased significantly, particularly with teriflunomide, CdA and anti-CD20. The percentage of CD20^+^CD27^+^ B cells increased with NTZ and teriflunomide. Following anti-CD20 treatment, the percentage of CD20^-^CD27^+^ B cells increased markedly compared to the other therapies (**Supplementary Table S5**, **Figure 3A**). Once again, anti-CD20 demonstrated the most pronounced differences relative to other treatments in the expression of the CD40, HVEM and PD-1 molecules in CD20^+^CD27^-^ B cells. Lastly, BTLA expression in CD20^+^CD27^+^ B cells was higher after CdA compared to DMF (**Supplementary Table S5**, **Figure 3B**).

Regarding T cells, DMF treatment resulted in significantly higher CTLA-4 expression on effector Th cells compared to CdA, NTZ and teriflunomide treatment. In addition, after DMF, HVEM expression in memory Th cells is diminished in comparison with teriflunomide and CdA. Finally, PD-1 expression in memory CD8^+^ T cells decrease after teriflunomide and NTZ compared to DMF and anti-CD20 treatment (**Supplementary Table S5**, **Figure 3C**).

Finally, within the innate immune system, treatment-related differences were also observed, particularly an increased expression of PD-L1 on NK^dim^ cells following teriflunomide treatment (**Supplementary Table S5**, **Figure 3C**).

### 3.4 Immune profiling as a predictor of relapse risk, MRI activity, and disability progression

Clinical relapses, new MRI lesions, and disability progression do not necessarily occur simultaneously, as an event indicating lack of response may arise independently of the others. Therefore, we identified immunological biomarkers for the presence of each individual clinical variable.

**Figure 4:**
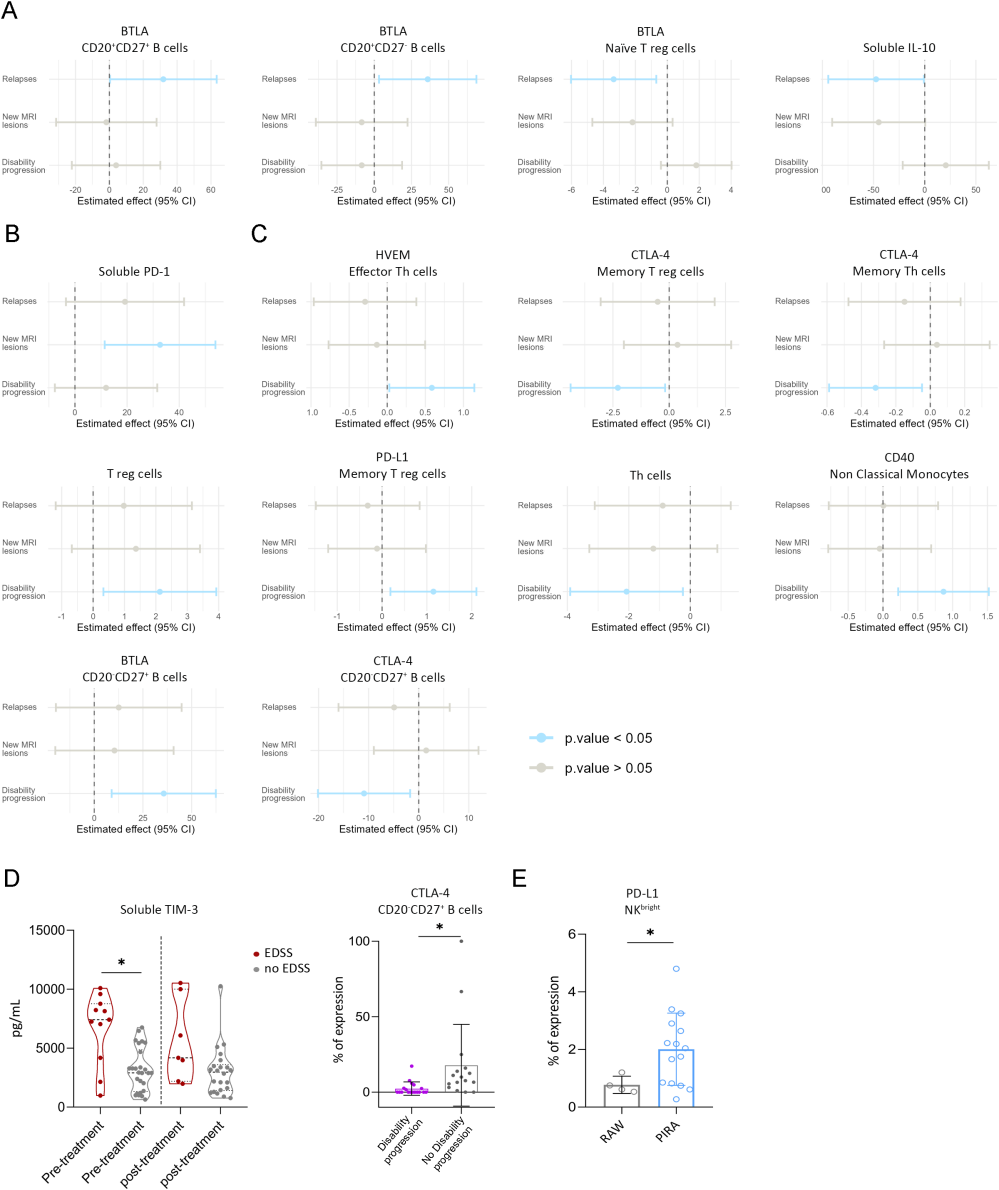
Immune biomarkers for relapse, MRI activity, and disability progression. **(A–C)** Linear regression analysis of immune cell populations and immune checkpoint expression with clinical variables associated with non-responders. Plasma soluble ICs and cytokines are included **(A)** Associations with relapses; **(B)** Associations with New MRI lesions; **(C)** Associations with disability progression; **(D)** Disability progression molecules assessed with EDSS. Plasma of soluble TIM-3 concentration across the study in patients with disability progression (red dots) and no disability progression (grey dots). CTLA-4 expression on CD20^-^CD27^+^ B cells showing differences between disability progression and patients with stable disease; **(E)** Percentages of PD-L1 expression on NK^bright^ cells between patients showing PIRA (blue dots) and patients showing RAW (grey dots) Data are presented as mean ± SD. *p < 0.05. EDSS = Expanded Disability Status Scale. Blue bars represent significant correlations. NK=Natural Killer. Relapse (n) = 12; new MRI lesions (n)=24; disability progression (n)=28; PIRA (n)=22; RAW (n)=6.

In patients who experienced a relapse during the 12 months of follow up, an increase of BTLA expression in CD20^+^CD27^+^ and CD20^+^CD27^-^ B cells, a decrease of BTLA in naïve T reg and a decrease in soluble IL-10 in plasma concentration was observed at three months post-treatment initiation (estimate = −47.54; 95% CI: −94.27 to −0.80; *p* = 0.047) (**Figure 4A**; **Supplementary Table S6**).

Patients who developed new MRI lesions exhibited significantly higher plasma levels of soluble PD-1 at three months post-treatment compared to those without new lesions (estimate = −32.65; 95% CI: −11.41 to −53.89; *p* = 0.004) (**Figure 4B**).

Regression analysis revealed that lower CTLA-4 expression on CD20^-^CD27^+^ B cells was significantly associated with disability progression (estimate = −10.93; 95% CI: −20.13 to −1.72; *p* = 0.022). Similar inverse associations were observed for CTLA-4 expression on memory regulatory T cells (estimate = −2.28; 95% CI: −4.38 to −0.18; *p* = 0.034) and memory Th cells (estimate = −0.32; 95% CI: −0.59 to −0.05; *p* = 0.023). Conversely, HVEM expression on effector Th cells showed a positive association with disability progression (estimate = 0.59; 95% CI: 0.03 to 1.14; *p* = 0.040) (**Figure 4C**). Consistently, patients who experienced disability progression during follow-up showed significantly lower CTLA-4 expression on CD20^-^CD27^+^ B cells compared to those who did not progress (2.36 ± 4.42% vs. 17.87 ± 27.06%; *p* = 0.039) at three months after treatment initiation (**Figure 4D**). In addition, soluble plasma TIM-3 pre-treatment levels in plasma were higher in patients who exhibited disability progression (6714.11 ± 2988.63 pg/mL and 3153.6 ± 1827.92 pg/mL; *p* = 0.035) (**Figure 4D**). This finding suggests a pre-existing pro-activating immune environment in patients who go on to develop disability worsening progression.

### 3.5 PD-L1 on NK^bright^ cells indicates PIRA and RAW in MS patients

We evaluated whether immune cell distribution and IC expression at three months post-treatment could predict RAW and PIRA at 12 months. Notably, patients who developed PIRA exhibited significantly higher PD-L1 expression on NK^bright^ cells compared to those with RAW (2.01 ± 1.25% vs. 0.77 ± 0.3%; *p* = 0.036) (**Figure 4E**).

### 3.6 CD70 expression on Natural Killer^bright^ predicts early treatment response

**Figure 5:**
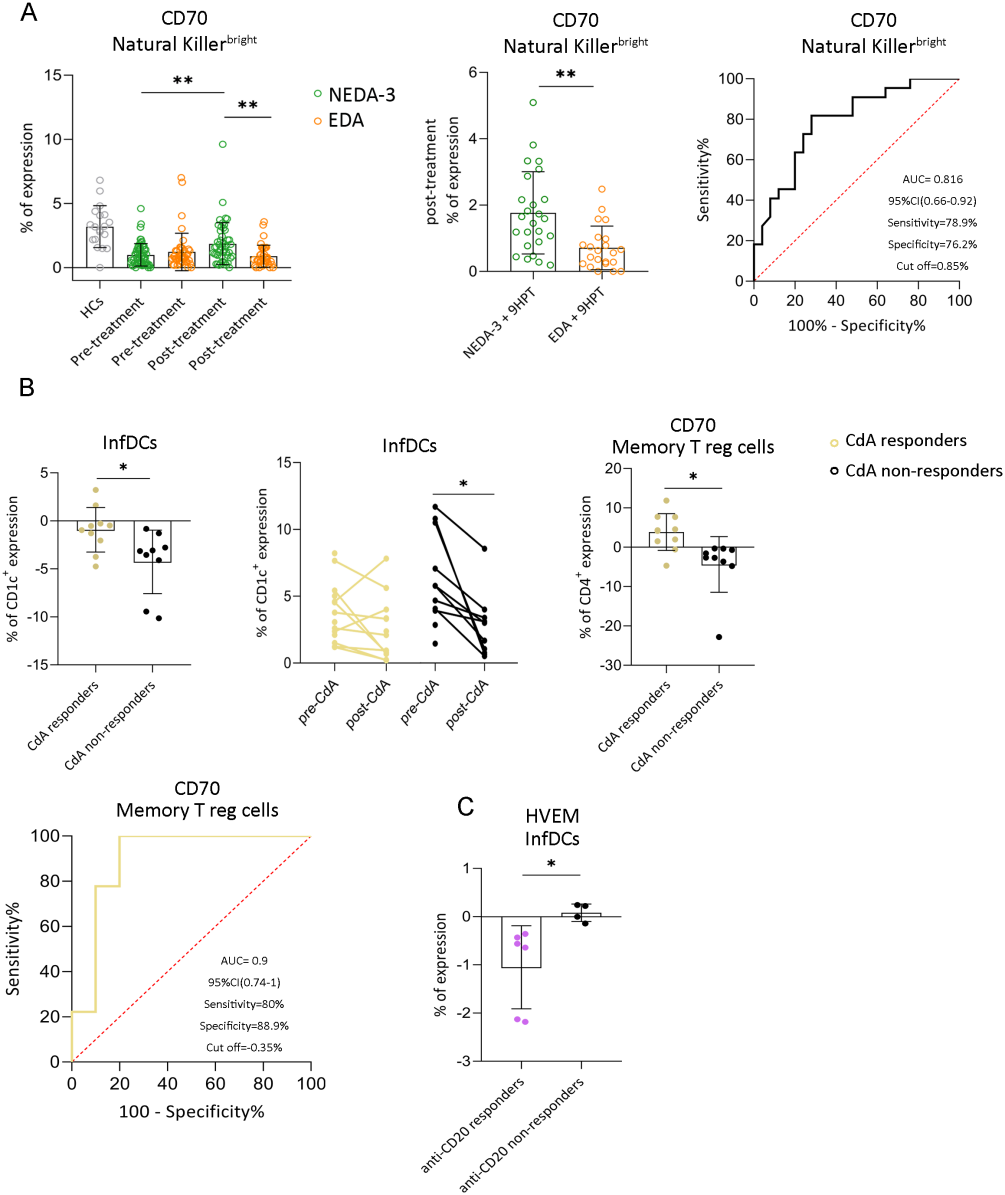
Immune phenotype that predicts response to treatment. **(A)** Differences in CD70 expression in NK^bright^ percentages between responders and non-responders throughout the study, both pre-treatment and post-treatment data. CD70 on NK^bright^ expression levels in healthy controls (grey dots), responders (green dots) and non-responders (orange dots). ROC curve analysis revealed an AUC of 0.816, with a sensitivity of 78.9% and specificity of 76.2%, using a cut-off of 0.85% of CD70 expression on NK^bright^ at 3 months post-treatment (black curve). NEDA-3=No Evidence of Disease Activity; EDA=Evidence of Disease Activity; 9HPT=Nine-Hole Peg Test; NK=Natural Killer. **(B)** Patients treated with CdA. Comparison of IC expression and immune subsets before treatment and at three months after treatment between responders (left dots) and non-responders (right dots). CD70 expression on memory Treg cells was significantly lower in non-responders compared to responders 3 months post CdA-treatment, ROC analysis revealed that CD70^+^ memory Treg frequency had strong predictive power for response to CdA treatment (AUC = 0.9). A threshold of -0.35% provided 80% sensitivity and 88.9% specificity (yellow curve); **(C)** Patients treated with anti-CD20. NEDA + 9HPT (n)=39; EDA + 9HPT (n)=32; CdA responders (n)=16; CdA non-responders (n)=16; Anti-CD20 responders (n)=13; Anti-CD20 non-responders (n)=10. Analyses were performed across all treatments; only therapies showing statistically significant differences (CdA and anti-CD20) are shown. Data are presented as mean ± SD. *p < 0.05. **p < 0.01.

Using the NEDA-3 criteria, which evaluate relapses, MRI lesions, and disability progression together, we looked for an early biomarker to identify responders and non-responders to treatment. Responders exhibited a marked increase in expression of CD70 on NK^bright^ at three months post-treatment compared to their pre-treatment values (1.03 ± 0.92% and 2.08 ± 1.74%; *p* = 0.002). Moreover, non-responders showed significantly lower CD70 expression compared to responders (0.86 ± 0.8% and 1.98 ± 1.7%; *p* = 0.002). This difference in expression levels and statistical significance remained consistent even when incorporating a more stringent definition of treatment response by adding motor disability progression assessed by the 9HPT (0.71 ± 0.66% and 1.77 ± 1.24%; *p* = 0.008). ROC curve analysis revealed an AUC of 0.816, with a sensitivity of 78.9% and specificity of 76.2%, which identify a cut-off of 0.85% of CD70 expression that differentiate responders from non-responders (**Figure 5A**).

### 3.7 Therapy-specific immune biomarkers of treatment response

Given the distinct immunological targets of each therapy, we examined immune responses three months after treatment initiation to identify cell-specific biomarkers predictive of treatment response at 12 months post-treatment initiation for each therapeutic approach.

At three months post-treatment with CdA, responders showed increased expression of CD70 and PD-L1 on NK^dim^ and CD20^+^CD27^-^ B cells, along with a decrease in naïve CD8+ T cells, and in CD20^+^CD27^-^, CD20^+^CD27^+^ and total B cell populations (**Supplementary Table S7**). A slight increase in CD70 expression on memory Treg cells was also observed in responders compared to non-responders (3.85 ± 4.67% vs. -4.38 ± 7.07%, *p* = 0.017). In contrast, non-responders exhibited a post-treatment reduction in naïve Treg cells and a significant decrease in InfDC frequency (7.14 ± 3.06% vs. 2.89 ± 2.48%, *p* = 0.015), which was more pronounced than in responders (-4.26 ± 3.31% vs. -0.92 ± 2.32%, *p* = 0.027) (**Figure 5B**, **Supplementary Table S7**). Interestingly, a slight increase in the CD70 expression on memory T reg cells was observed in responders compared to non-responders (3.85 ± 4.67% and -4.38 ± 7.07%; *p* = 0.017). A decrease in CD70 expression on memory Treg cells of less than -0.35% from pre to three months post-treatment was identified by ROC analysis as the most discriminative threshold for treatment response (sensitivity: 80%, specificity: 88.9%, AUC = 0.9). That is, a minimal reduction or stable expression of CD70 on memory Treg cells serves as an indicator of therapeutic response to CdA (**Figure 5B**).

At three months post-treatment, patients responding to anti-CD20 therapies exhibited a significant reduction in total B cells, including the CD20^+^CD27^-^ B cells subset, as well as a marked decrease in CD80 expression on CD20^+^CD27^+^ B cells compared to baseline (**Supplementary Table S7**). Furthermore, non-responders to anti-CD20 treatment showed significantly higher levels of HVEM expression on InfDCs compared to responders (0.08 ± 0.18% vs. -1.05 ± 0.86%, *p* = 0.029) (**Figure 5C**).

In NTZ responders, an increase in HVEM expression on intermediate monocytes was observed at three months relative to pre-treatment levels (**Supplementary Table S7**).

### 3.8 Correlation of ligand-receptor interactions in ICs in responders and in non-responders

We analyzed the correlation between IC ligand-receptor pairs across the various cell populations (**Table 2**). We observed that BTLA ligand expression in CD20^-^CD27^+^ B cells, naïve Th cells, naïve T reg cells, naïve CD8^+^ T cells, InfDCs, and NK positively correlated with HVEM expression in effector Th cells in responder patients suggesting an inhibitory effect of HVEM in responder group at 3 months post-treatment. Moreover, CTLA-4 expressed in memory Th cells showed a significant correlation with its ligand CD80 in CD20^-^CD27^+^ B cells in both responders and non-responders, which supports the robustness of our results.

**Table 2:** Correlations between IC ligands and receptors in responders versus non-responders post-treatment.

	Responders		Non-responders	
Correlation L-R	HVEM in effector Th cells			
BTLA expression	R	*p*-value	R	*p*-value
CD20^+^CD27^-^ B cells	0.059	0.691	0.262	0.170
CD20^+^CD27^+^ B cells	0.245	0.094	0.220	0.252
CD20^-^CD27^+^ B cells	0.510	0.000*	0.249	0.193
Naïve Th cells	0.386	0.007*	-0.056	0.772
Naïve T reg	0.452	0.001*	0.545	0.002*
Naïve CD8^+^ T cells	0.403	0.005*	0.006	0.975
InfDCs	0.526	0.000*	0.273	0.152
Natural killer ^Bright^	0.359	0.012*	-0.298	0.117
Natural killer ^dim^	0.592	0.000*	-0.008	0.969
	CTLA-4 in memory Th cells			
CD80 expression	R	*p*-value	R	*p*-value
CD20^+^CD27^+^ B cells	0.274	0.060	0.096	0.622
CD20^-^CD27^+^ B cells	0.482	0.001*	0.542	0.002*
Memory T reg	-0.046	0.758	0.105	0.587

*Speraman or Pearson tests were employed to determine statistically significant between groups. L, ligand; R, receptor; H, helper.Bold means *p* < 0.05.

## 4 Discussion

In this study, we analyzed immune subpopulation proportions and their IC expression in the search for a therapeutic response biomarker. We identified differential IC expression patterns between MS patients and HCs, revealing specific ICs involved in the disease. Within the MS cohort, we observed differences between treatment responders and non-responders. Responders exhibited higher CD70 expression on NK^bright^ cells post-treatment. Notably, CD70 expression on memory Treg cells emerged as a minimally invasive early biomarker for response to Cladribine. Additionally, we propose that elevated inhibitory CTLA-4 levels on CD20^-^CD27^+^ B cells may serve as a protective factor against disability progression. BTLA expression on CD20^+^CD27^-^ B cells was associated with relapse events in patients, and PD-L1 expression on NK^bright^ cells appeared to be a potential biomarker for PIRA. Beyond biomarker identification, this study also evaluated immune system behaviour under different therapies by analyzing paired pre- and post-treatment samples. This longitudinal approach allowed us to characterize treatment-induced immunological changes and link them to clinical outcomes. Importantly, we found robust associations between key indicators of treatment failure, including relapses, new MRI lesions, and disability progression and the expression of both membrane and soluble ICs, as well as plasma cytokine levels.

Spectral flow cytometry is a powerful tool that has gained significant traction in recent years, though its integration into routine clinical practice is still under development. Several studies have employed spectral flow cytometry to characterize specific immune cell profiles and their association with disease activity. For example, circulating lymphocyte subsets in treatment-naïve individuals with MS and other CNS autoimmune diseases have been analyzed (30). Additionally, receptor expression in treatment-naïve MS patients has been monitored longitudinally to track changes throughout therapy (31). Furthermore, subtle longitudinal immune alterations characteristic of therapeutic response have been investigated using this technique (32).

With this main idea, the first step in this study was to compare IC expression and the proportion of immune populations between HCs and MS patients. The results demonstrated significant differences in the expression of co-inhibitory and co-stimulatory molecules and cell population proportions between both groups, suggesting an altered immune profile in MS patients. Some ICs, such as PD-1, CD40, BTLA, HVEM, and CD28, exhibited both increased and decreased expression depending on the immune cell subset, indicating a cell-type-specific regulation. Despite this variability, these IC were more frequently upregulated in MS patients across the analyzed populations, and were therefore classified as predominantly increased in MS. In contrast, CTLA-4 and CD70 consistently showed reduced expression in multiple immune subsets in MS, supporting their classification as predominantly decreased in MS patients. These patterns suggest a context-dependent immune dysregulation that varies by IC and cell type. Dysregulation of IC molecules within the synapse can lead to excessive activation of autoreactive cells, ultimately triggering an inflammatory cascade that results in demyelination and neurodegeneration (5). Specifically in lymphoid lineage, our findings reveal altered IC expression on CD20^+^CD27^+^, CD20^-^CD27^+^, CD20^+^CD27^-^ B cells in MS patients compared to HCs, suggesting a disrupted immune environment that affects B cell activation. Particularly in CD20^+^CD27^+^ B cells, CD80 expression was increased in MS patients, suggesting enhanced immune activation given the co-stimulatory role of this molecule. Within the CD20^-^CD27^+^ B cells population, PD-L1 and CTLA-4 were significantly downregulated in MS patients compared to HCs, indicating a reduced capacity to suppress the immune system, consistent with the known inhibitory functions of these ICs. Our results align with previous research demonstrating B cell dysfunction in MS (33). Th cells revealed differences in IC expression, particularly lower PD-1 expression on effector and memory Th cells and lower CTLA-4 on memory Th cells in MS patients. In CD8^+^ T cells, memory CD8^+^ T cells from MS patients showed increased of the co-stimulatory molecule CD28, indicating a potentially heightened activation state within this subset. MS patients showed reduced co-inhibitory PD-1 on terminal effector CD8^+^ T cells. These results suggest that peripheral T cells may be subjected to insufficient negative regulation. These observations are consistent with previous studies demonstrating distinct PD-1 expression patterns in immune cell subsets of RRMS patients compared to HCs (34). Myeloid cells, including monocytes and DCs, exhibited significant differences in IC expression, further highlighting their involvement in the immunopathogenesis of MS. The increased expression of CD40 and HVEM on classical and intermediate monocytes in MS patients suggests heightened activation of these cells, which could enhance the antigen-presenting capabilities of myelin proteins and promote T cell activation (35). In this regard, previous studies agreed with these results by linking genetic predisposition to modifications in the HVEM gene in MS patients (36). Similarly, in this study, InfDCs displayed elevated expression of HVEM, CD40 and BTLA. These findings align with previous studies demonstrating the function of CD40 expression on DCs for priming pathogenic Th cells and facilitating their migration to the CNS in animal models of MS (37). The observed upregulation of IC molecules in monocytes and InfDCs reinforces the idea that myeloid cells serve as key drivers of inflammation in MS by bridging innate and adaptive immune responses.

This study also reveals that DMTs for MS modify IC expression and immune cell populations. These findings provide valuable insights into the immunomodulatory mechanisms of these treatments, highlighting their distinct impacts on key cellular and molecular pathways involved in MS pathogenesis. Among the therapies analyzed, anti-CD20 demonstrated the most profound effects on B cell populations. It significantly reduced total B cell counts, CD20^+^CD27^+^ B cells and ICs expression, such as HVEM and PD-1 and CD40, compared to other DMTs. Interestingly, the percentage of CD20^-^CD27^+^ B cells increased markedly following anti-CD20 treatment, suggesting a shift in B cell subsets in patients receiving this treatment. This aligns with prior studies that indicate anti-CD20 selective deplete CD20 expressing B cells while sparing antibody-secreting CD20^-^CD27^+^ B cells (38). DMF also notably altered IC expression, with significant reductions in HVEM on memory Th cells. These findings support prior reports highlighting DMF ability to switch T cell populations toward anti-inflammatory phenotypes, including regulatory subsets (39, 40). For its part, teriflunomide induced a decrease in PD-1 expression on memory CD8^+^ T cells compared to DMF, suggesting differing mechanisms of action between these therapies in modulating cytotoxic T cell subsets. These findings complement previous research showing teriflunomide immunomodulatory effects through the inhibition of pyrimidine synthesis, leading to reduced T cell proliferation and cytokine production (41). Despite the numerous studies using similar approaches, we found it relevant to explore immune fluctuations in relation to treatment within our cohort. These findings emphasise the differential impacts of DMTs on IC pathways and immune cell populations, highlighting the importance of tailoring treatments to the specific immunological profiles of MS patients.

As an additional step, we performed a sub-analysis by treatment to identify the immune biomarkers that specifically reflected therapeutic response for each individual treatment. In the case of CdA, its mechanism of action involves the depletion of B and T cells through apoptosis, leading to a temporary reduction in immune activation. This depletion is followed by a gradual immune system reconstitution, promoting a homeostatic balance of immune cells (42) we observe the principal T cells decrease in responders as well as reconstitution of CD20^+^CD27^-^ B cells and Treg cell subsets after cladribine (43). The observed increase in InfDCs in responders could reflect an adaptive immune response post-treatment, facilitating better immune regulation and reduced inflammation. Additionally, the increased proportion of CD70 on memory Treg in CdA responders suggests a more balanced immune response. Regulatory T cells suppress immune responses and thus contribute to immune homeostasis. Among the many mechanisms implied in Treg-mediated suppression, the inhibition of InfDCs has been shown to be central in peripheral tolerance induction (44). Based on our findings, we propose the expression of CD70 on memory Tregs as a potential biomarker of therapeutic response to Cladribine. Moreover, patients responding to anti-CD20 therapies exhibited a significant reduction in CD20^+^CD27^-^ B cell subset and CD80 expression on CD20^+^CD27^+^ B cells compared to baseline and responders to NTZ showed an increase in HVEM expression on intermediate monocytes. These results underscore the potential of various cell surface molecules as biomarkers for predicting response to different immunological treatments. While further validation in larger cohorts is necessary, these findings provide valuable insights into how these therapies modulate the immune system and how biomarkers can guide personalised treatment strategies.

Although no specific biomarker of response to treatment could be identified for the remaining therapies, possibly due to sample size, we were able to identify a biomarker of treatment response that appears to be shared across all treatment groups. We identified immune-based biomarker that predict treatment response: CD70 expression on NK^bright^ cells at 3 months post-treatment. NK cells may reduce disease progression by suppressing pathogenic T cells (45) and reducing disease activity. Additionally, CD70 expression on NK cells showed low cytotoxicity (46) and because of their immunomodulatory properties, the CD56^bright^ subset is suggested to have beneficial effects on the MS disease course (47). This finding regarding CD70 expression in both memory regulatory T cells and NK^bright^ cells aligns with our results in MS patients compared to HCs. Three months post-treatment, responders displayed higher levels of CD70, resembling the expression pattern observed in HCs. This suggests that the acquisition of a “healthy-like” immune phenotype may serve as a favourable prognostic marker.

In a further subanalysis, we investigated the immunological mechanisms underlying relapses, MRI activity, and disability progression measured by EDSS. Patients who experienced relapses during follow-up exhibited increased BTLA expression on CD20^+^CD27^-^ B cells. Although BTLA is typically considered an inhibitory receptor, this upregulation may reflect a compensatory, yet ultimately insufficient, attempt to regulate disease activity, consistent with previous studies linking BTLA expression on B cells with modulation of MS severity (48). In parallel, higher IL-10 levels were associated with lower relapse rates, highlighting the anti-inflammatory role of this cytokine in suppressing activated macrophages and DCs and in regulating innate and adaptive immune responses (49). These findings support the idea that relapse events are strongly influenced by imbalances in regulatory B cell function and cytokine-mediated control.

Regarding MRI activity, associations were observed with soluble PD-1 levels, consistent with prior reports (34), indicating that this marker may reflect ongoing subclinical disease activity. These results point to distinct immune mechanisms underlying MRI-detected inflammation, which may not always coincide with overt clinical relapses.

Progression of disability, measured by EDSS, was associated with lower CTLA-4 expression in CD20^-^CD27^+^ B cells, suggesting that reduced CTLA-4 levels may contribute to enhanced plasmablast activation and autoantibody production, thereby promoting inflammation during silent disease progression (50). Furthermore, a decrease in Th cell frequency was linked to the absence of EDSS worsening, aligning with the established pathogenic role of Th cells in MS (51). Increased HVEM expression in effector Th cells was also observed in patients with disability progression, although no correlation with BTLA was detected suggesting that HVEM may act through activating pathways, potentially involving other ligands such as LIGHT (52). PD-L1 expression on memory Treg cells was additionally associated with disability progression, which could indicate an ineffective regulatory environment in patients experiencing worsening of neurological function. In patients with PIRA, we observed increased PD-L1 expression on NK^bright^ cells compared to those with RAW. This suggests that PD-L1 on NK cells may play a role in limiting acute inflammatory episodes, potentially acting as a protective factor in this clinical phenotype (18). Whether this expression reflects effective immune regulation or an exhausted activation profile remains to be determined, but it highlights the complexity and specificity of immune mechanisms across MS subtypes.

Notably, the correlations observed between ligand-receptor interactions, particularly BTLA-HVEM and CTLA-4-CD80, provide mechanistic insights into immune regulation in response to treatment. The significant correlations between these molecular interactions further support our findings, reinforcing the idea that these pathways play a crucial role in shaping therapeutic responses.

Overall, these results underscore the complexity of the immune profile in non-responder patients and highlight the need to consider each clinical variable independently to better understand the pathogenesis. One limitation of the study is that the study’s sample size was calculated to detect differences between responders and non-responders, which resulted in reduced power for analyses within individual treatment groups and particularly small numbers in some treatment subgroups; these subgroup findings should therefore be interpreted with caution. Another limitation of our study is that serum biomarker assessment was restricted to a single time point, at three months after treatment initiation. This approach may not fully capture the delayed pharmacodynamic effects of certain disease-modifying therapies, such as teriflunomide, which are considered to exert their maximal effect only after a longer period. In addition, some DMTs, such as fingolimod or natalizumab, may carry a risk of disease reactivation upon withdrawal, even when the recommended washout period is observed, potentially creating a misleading impression of treatment failure when a new therapy is introduced. Nevertheless, the study design was intentional as our primary aim was to identify early biomarkers capable of predicting treatment response or failure once the patient is already under the pharmacological effect of the new therapy. For this reason, immune biomarkers were assessed at three months, while clinical and MRI response or failure was evaluated at 12 months, a time point chosen in line with the ECTRIMS consensus guidelines (25) and when residual effects of the previous therapy are no longer expected to influence outcomes. Detecting treatment failure at an earlier stage is of particular relevance, since disability progression after 12 months of ineffective therapy may already be irreversible.

This study offers a comprehensive characterization of IC expression and immune cell subpopulation dynamics in patients with MS, underscoring their potential role in modulating treatment response and disease progression. Our data suggest that relapses are linked to increased BTLA expression in CD20^+^CD27^+^ and CD20^+^CD27^-^ B cells, decreased BTLA in naïve Treg, and lower plasma IL-10, suggesting insufficient regulatory control. MRI activity was associated with higher soluble PD-1 levels, reflecting ongoing subclinical inflammation. Disability progression corresponded to reduced CTLA-4 in CD20^-^CD27^+^ B cells, memory Treg, and Th cells, along with increased HVEM in effector Th cells and elevated pre-treatment soluble TIM-3, indicating a pro-activating immune environment. According to NEDA-3 criteria, responders exhibited early upregulation of CD70 on NK^bright^ cells, whereas non-responders showed lower expression, supporting its potential as a predictive biomarker. These findings highlight the clinical relevance of IC profiling and immune cell phenotyping as promising early biomarkers for predicting treatment response.

## Data Availability

The raw data supporting the conclusions of this article will be made available by the authors, without undue reservation.
