# Supplementary material for: Immune checkpoint-based biomarkers for therapeutic response in patients with multiple sclerosis
Source: Front Immunol. 2025 Oct 20;16:1694021. doi: 10.3389/fimmu.2025.1694021 (PMC12580306; doi:10.3389/fimmu.2025.1694021)

Supplementary Material

# Supplementary Tables

**Supplementary Table S1.** **List of antibodies used for immune phenotyping**

| **Antibody** | **Fluorochrome** | **Clone** | **Vendor** | **Catalogue number** |
| --- | --- | --- | --- | --- |
| HLADR MHC Class II | BUV496 | Tu39 | BD Bioscience (San Jose, CA, USA) | 741157 |
| CD27 | BUV563 | L128 | BD Bioscience (San Jose, CA, USA) | 748705 |
| CD80 | BUV661 | 2D10.4 | BD Bioscience (San Jose, CA, USA) | 751731 |
| CD70 | BV786 | Ki-24 | BD Bioscience (San Jose, CA, USA) | 565338 |
| HVEM | BV480 | CW10 | BD Bioscience (San Jose, CA, USA) | 746607 |
| CD4 | BUV805 | RPA-T4 | BD Bioscience (San Jose, CA, USA) | 742000 |
| CD40 | BUV737 | 5C3 | BD Bioscience (San Jose, CA, USA) | 741847 |
| CD127 | APC-R700 | HIL-7R-M21 | BD Bioscience (San Jose, CA, USA) | 565185 |
| CD45RA | BUV395 | HI100 | BD Bioscience (San Jose, CA, USA) | 740298 |
| PD-1 | BV421 | NAT105 | BioLegend (San Diego, CA, USA) | 367422 |
| CD25 | PE-Fire 700 | M-A251 | BioLegend (San Diego, CA, USA) | 356145 |
| CD28 | BV605 | CD28.2 | BioLegend (San Diego, CA, USA) | 302968 |
| PDL1 | BV711 | [29E.2A3](https://www.biolegend.com/en-gb/search-results?Clone=29E.2A3) | BioLegend (San Diego, CA, USA) | 329722 |
| CD3 | Spark Blue 550 | SK7 | BioLegend (San Diego, CA, USA) | 344852 |
| BTLA | PE-Dazzle 594 | MIH26 | BioLegend (San Diego, CA, USA) | 344522 |
| CTLA-4 | Alexa Fluor 647 | BNI3 | BioLegend (San Diego, CA, USA) | 369626 |
| CD19 | APC-Fire 810 | HIB19 | BioLegend (San Diego, CA, USA | 302272 |
| CD8 | cFluor V547 | SK1 | Cytek Biosciences Inc., Fremont, CA, USA | R7-20063 |
| CD20 | cFluor B675 | 2H7 | Cytek Biosciences Inc. (Fremont, CA, USA) | R7-20195 |
| CD14 | cFluor V450 | M5E2 | Cytek Biosciences Inc. (Fremont, CA, USA) | R7-20003 |
| CD56 | cFluor BYG750 | LT56 | Cytek Biosciences Inc. (Fremont, CA, USA) | R7-20283 |
| CD1C | PerCP-eFluor 710 | L161 | Invitrogen™, Thermo Fisher Scientific (Waltham, MA, USA) | 46-0015-42 |
| CD16 | Alexa Fluor 488 | 3G8 | STEMCELL Technologies Inc., (Vancouver, BC, Canada) | 60041AD |
| OX40 | PE | 443318 | Invitrogen™, Thermo Fisher Scientific (Waltham, MA, USA) | MA5-23676 |
| Viability Live dead | UV488 |  | Invitrogen™, Thermo Fisher Scientific (Waltham, MA, USA) | L34961 |

**Supplementary Table S2. ICs Geometric Mean Fluorescence Intensity (MFI)**

|  | **HCs**  **(mean±SD)** | **Responders**  **(mean±SD)** | **Non-responders**  **(mean±SD)** |
| --- | --- | --- | --- |
| CD20^+^ CD27^-^ B cells BTLA | 49213,95 ± 17613,65 | 47751,57 ± 26719,17 | 39732,72 ± 23099,59 |
| CD20^+^ CD27^-^ B cells CD40 | 51498,68 ± 7310,84 | 41898,84 ± 19454,43 | 38749,2 ± 16546,34 |
| CD20^+^ CD27^-^ B cells HVEM | 14870,79 ± 1988,62 | 28536,24 ± 22514,6 | 35574,88 ± 27527,06 |
| CD20^+^ CD27^-^ B cells  OX40 | 5082,05 ± 813,48 | 4832,97 ± 3507,41 | 4350,2 ± 3332,98 |
| CD20^+^ CD27^-^ B cells  PD-1 | 4258,79 ± 4859,28 | 5872,51 ± 2828,85 | 6273,68 ± 2852,87 |
| CD20^+^ CD27^+^ B cells BTLA | 40303,32 ± 10366,01 | 31532,21 ± 16211,6 | 29028,19 ± 14744,26 |
| CD20^+^ CD27^+^ B cells CD40 | 62556,42 ± 8427,49 | 53100,86 ± 18709,09 | 50587,32 ± 19487,96 |
| CD20^+^ CD27^+^ B cells CD70 | 10990,79 ± 4193,5 | 8893,33 ± 5364,89 | 7302,53 ± 3879,54 |
| CD20^+^ CD27^+^ B cells CD80 | 11582,42 ± 2508,51 | 10295,54 ± 7428,77 | 9241,5 ± 7549,9 |
| CD20^+^ CD27^+^ B cells CTLA-4 | 3258,16 ± 874,45 | 3993,37 ± 2332,12 | 3983,11 ± 2019,86 |
| CD20^+^ CD27^+^ B cells HVEM | 21018,05 ± 4854,31 | 17152,06 ± 8198,91 | 14778,0 ± 6443,73 |
| CD20+ CD27+ B cells PD-L1 | 7783,84 ± 2330,74 | 9097,33 ± 5811,66 | 8910,58 ± 6232,49 |
| CD20^-^ CD27^+^ B cells BTLA | 56351,44 ± 22478,7 | 34343,61 ± 19432,17 | 29937,0 ± 21325,1 |
| CD20^-^ CD27^+^ B cells CD40 | 60662,44 ± 22346,64 | 46910,25 ± 21813,2 | 48640,37 ± 21801,74 |
| CD20^-^ CD27^+^ B cells CD70 | 7336,06 ± 3239,68 | 4548,71 ± 2535,98 | 4017,13 ± 2675,24 |
| CD20^-^ CD27^+^ B cells CD80 | 10288,08 ± 5268,47 | 9707,09 ± 5749,51 | 9429,87 ± 5080,37 |
| CD20^-^ CD27^+^ B cells CTLA-4 | 3595,24 ± 1311,51 | 3294,04 ± 1649,0 | 2973,9 ± 1563,89 |
| CD20^-^ CD27^+^ B cells HVEM | 13938,6 ± 7643,95 | 10427,81 ± 6033,19 | 11174,32 ± 6215,82 |
| CD20^-^ CD27^+^ B cells  PD-L1 | 6851,53 ± 3505,28 | 8572,97 ± 4549,02 | 8699,35 ± 4593,05 |
| Effector Th  CD28 | 25925,79 ± 5564,71 | 13600,93 ± 8148,49 | 9996,24 ± 4843,06 |
| Effector Th  CTLA-4 | 5058,0 ± 1987,92 | 2969,55 ± 1005,94 | 2876,73 ± 1053,23 |
| Effector Th  HVEM | 14587,5 ± 4347,65 | 11380,68 ± 6363,9 | 11358,07 ± 8292,74 |
| Effector Th  OX40 | 7632,19 ± 3641,61 | 6099,39 ± 3482,95 | 5459,18 ± 2902,38 |
| Effector Th  PD-1 | 6154,11 ± 3275,97 | 11166,07 ± 11608,87 | 10140,88 ± 6697,47 |
| Effector Th  PD-L1 | 11850,37 ± 4006,22 | 10014,36 ± 5507,42 | 10898,62 ± 8740,39 |
| Memory Th  CD28 | 24991,68 ± 4423,48 | 12616,63 ± 8895,69 | 9131,06 ± 5498,46 |
| Memory Th  CTLA-4 | 4623,21 ± 694,83 | 3586,15 ± 1080,33 | 4091,27 ± 1613,65 |
| Memory Th  HVEM | 17490,26 ± 4973,56 | 11877,81 ± 7144,18 | 11406,85 ± 8901,38 |
| Memory Th  OX40 | 10591,61 ± 4530,91 | 8021,44 ± 5256,99 | 6590,15 ± 4223,91 |
| Memory Th  PD-1 | 5352,11 ± 1071,74 | 9634,91 ± 6631,36 | 10051,68 ± 7329,59 |
| Memory Th  PD-L1 | 11811,21 ± 3480,2 | 11221,88 ± 3850,84 | 11408,78 ± 4580,58 |
| Naïve Th  BTLA | 32461,06 ± 11346,65 | 25825,95 ± 11809,94 | 23230,62 ± 9621,2 |
| Naïve Th  CD28 | 14586,58 ± 2339,64 | 7230,72 ± 4730,66 | 5415,18 ± 3430,55 |
| Naïve Th  HVEM | 16283,28 ± 5434,74 | 11701,74 ± 6740,21 | 10459,77 ± 6369,03 |
| Memory Treg  CD28 | 24302,26 ± 4014,35 | 11348,88 ± 8917,28 | 8148,21 ± 6150,95 |
| Memory Treg  CD70 | 5460,72 ± 1538,09 | 4869,17 ± 1849,41 | 5035,3 ± 1918,83 |
| Memory Treg  CD80 | 14122,19 ± 8092,07 | 10413,29 ± 6216,79 | 9817,71 ± 6680,77 |
| Memory Treg  CTLA-4 | 6497,41 ± 2484,79 | 3722,2 ± 1061,99 | 4014,1 ± 1623,13 |
| Memory Treg  HVEM | 19281,87 ± 9329,11 | 11355,39 ± 6690,08 | 10174,73 ± 6725,55 |
| Memory Treg  OX40 | 9437,25 ± 4203,29 | 8498,39 ± 5635,85 | 5869,79 ± 3640,64 |
| Memory Treg  PD-1 | 8199,82 ± 2900,04 | 9716,33 ± 4750,41 | 10099,68 ± 6315,3 |
| Memory Treg  PD-L1 | 22388,94 ± 16559,0 | 11572,51 ± 5676,25 | 10750,78 ± 5113,97 |
| Naïve Treg  BTLA | 22497,56 ± 7661,47 | 15951,89 ± 8589,22 | 14690,71 ± 9749,47 |
| Naïve Treg  CD28 | 14229,63 ± 2329,34 | 6153,23 ± 5732,53 | 4190,73 ± 4388,94 |
| Naïve Treg  CTLA-4 | 4137,94 ± 1458,63 | 1931,29 ± 739,32 | 1914,06 ± 703,99 |
| Naïve Treg  HVEM | 18750,56 ± 11010,63 | 11021,76 ± 5444,96 | 11280,27 ± 9569,59 |
| Naïve Treg  PD-L1 | 10217,22 ± 4944,23 | 9431,51 ± 3832,01 | 10291,17 ± 5729,89 |
| Effector CD8^+^ T cells CD28 | 15937,58 ± 4114,78 | 9423,71 ± 5826,39 | 7176,15 ± 3664,01 |
| Effector CD8^+^ T cells HVEM | 16686,47 ± 9354,47 | 11834,64 ± 7327,22 | 11229,43 ± 8394,53 |
| Effector CD8^+^ T cells PD-1 | 9990,0 ± 5249,81 | 10631,44 ± 5956,8 | 10726,97 ± 6421,18 |
| Effector CD8^+^ T cells PD-L1 | 9672,0 ± 4311,82 | 10078,87 ± 6949,1 | 9516,7 ± 7304,04 |
| Memory CD8^+^ T cells CD28 | 16324,16 ± 3601,11 | 9908,56 ± 5104,64 | 8254,18 ± 3294,0 |
| Memory CD8^+^ T cells HVEM | 19605,71 ± 9688,9 | 13975,83 ± 8422,15 | 12110,27 ± 7428,03 |
| Memory CD8^+^ T cells PD-1 | 10068,42 ± 5597,15 | 12528,79 ± 7421,79 | 12953,27 ± 8128,73 |
| Memory CD8^+^ T cells PD-L1 | 17182,17 ± 18625,89 | 11928,58 ± 6204,1 | 10167,09 ± 5294,14 |
| Naïve CD8^+^ T cells  BTLA | 26757,61 ± 8358,57 | 20615,98 ± 10703,45 | 19096,15 ± 9619,4 |
| Naïve CD8^+^ T cells  CD28 | 31513,42 ± 7392,12 | 13841,56 ± 10675,27 | 9795,18 ± 8247,47 |
| Naïve CD8^+^ T cells  HVEM | 15988,83 ± 5816,54 | 12491,44 ± 7122,56 | 10801,67 ± 6284,76 |
| Terminal effector CD8^+^ T cells  HVEM | 17489,72 ± 6894,6 | 12025,1 ± 6727,06 | 11053,52 ± 6695,7 |
| Terminal effector CD8^+^ T cells  PD-1 | 7443,06 ± 4888,68 | 10215,85 ± 6701,52 | 11318,64 ± 8042,04 |
| Terminal effector CD8^+^ T cells  PD-L1 | 10261,44 ± 4394,01 | 10117,98 ± 6668,52 | 10318,0 ± 7399,03 |
| InfDCS  BTLA | 32536,88 ± 14490,02 | 27304,44 ± 23449,61 | 26941,2 ± 24979,78 |
| InfDCS  CD40 | 101195,15 ± 54889,14 | 67494,35 ± 54463,6 | 44665,12 ± 45771,33 |
| InfDCS  HVEM | 35560,67 ± 19154,96 | 24554,3 ± 14418,8 | 20547,81 ± 13107,95 |
| Classical monocytes  CD40 | 99512,33 ± 33706,23 | 69838,29 ± 38995,9 | 61361,76 ± 30836,8 |
| Classical monocytes  HVEM | 38137,47 ± 14017,39 | 28712,95 ± 24112,22 | 26773,58 ± 23373,39 |
| Intermediate monocytes  CD40 | 124423,6 ± 59391,67 | 81391,14 ± 58987,8 | 72552,93 ± 51627,01 |
| Intermediate monocytes  HVEM | 41671,79 ± 20928,08 | 28770,36 ± 19927,65 | 24183,59 ± 14621,38 |
| Non-classical monocytes  CD40 | 110589,5 ± 63626,24 | 71252,1 ± 47238,98 | 59154,97 ± 35914,72 |
| Non-classical monocytes  HVEM | 36317,5 ± 17632,16 | 25235,47 ± 17946,72 | 21274,81 ± 13787,56 |
| NK^bright^  BTLA | 11460,0 ± 7513,77 | 8358,0 ± 4957,25 | 8226,0 ± 8644,2 |
| NK^bright^  CD70 | 2910,22 ± 864,52 | 6092,86 ± 4956,96 | 7123,2 ± 3074,83 |
| NK^bright^  HVEM | 14098,06 ± 5581,62 | 10649,4 ± 6501,58 | 8901,06 ± 5887,6 |
| NK^bright^  PD-1 | 4802,35 ± 3306,29 | 7535,41 ± 6158,35 | 8558,18 ± 7289,57 |
| NK^bright^  PD-L1 | 14580,17 ± 4736,84 | 11353,36 ± 3979,84 | 12068,59 ± 3964,15 |
| NK^dim^  BTLA | 11305,42 ± 3106,32 | 9678,05 ± 5665,08 | 9053,09 ± 6611,2 |
| NK^dim^  CD70 | 3671,0 ± 1682,89 | 4326,26 ± 1286,65 | 4250,71 ± 1446,97 |
| NK^dim^  HVEM | 15800,44 ± 4599,9 | 13246,14 ± 8058,9 | 11750,03 ± 7630,58 |
| NK^dim^  PD-1 | 4121,05 ± 1549,68 | 10735,42 ± 9440,88 | 12126,62 ± 10677,52 |
| NK^dim^  PD-L1 | 14299,32 ± 5607,34 | 12161,77 ± 6916,3 | 11108,91 ± 7972,55 |

**Supplementary Table S3. Cell counts.**

|  | **HCs**  **(max-min)** | **Responders**  **(max-min)** | **Non-responders**  **(max-min)** |
| --- | --- | --- | --- |
| B cells | 44459,0 - 862,0 | 34058,0 - 4,0 | 58969,0 - 1,0 |
| CD20^+^ CD27^-^ B cells | 34458,0 - 578,0 | 21402,0 - 0,0 | 44344,0 - 0,0 |
| CD20^+^ CD27^-^ B cells  BTLA | 12168,0 - 88,0 | 12092,0 - 0,0 | 6384,0 - 0,0 |
| CD20^+^ CD27^-^ B cells  CD40 | 34209,0 - 546,0 | 21226,0 - 0,0 | 44013,0 - 0,0 |
| CD20^+^ CD27^-^ B cells  HVEM | 345,0 - 13,0 | 146,0 - 0,0 | 204,0 - 0,0 |
| CD20^+^ CD27^-^ B cells  OX40 | 503,0 - 11,0 | 505,0 - 0,0 | 553,0 - 0,0 |
| CD20^+^ CD27^-^ B cells  PD-1 | 539,0 - 1,0 | 5826,0 - 0,0 | 3311,0 - 0,0 |
| CD20^+^ CD27^+^ B cells | 15824,0 - 247,0 | 13825,0 - 0,0 | 14898,0 - 0,0 |
| CD20^+^ CD27^+^ B cells BTLA | 2481,0 - 33,0 | 3712,0 - 0,0 | 5127,0 - 0,0 |
| CD20^+^ CD27^+^ B cells CD40 | 15411,0 - 217,0 | 13644,0 - 0,0 | 14070,0 - 0,0 |
| CD20^+^ CD27^+^ B cells CD70 | 1233,0 - 4,0 | 246,0 - 0,0 | 391,0 - 0,0 |
| CD20^+^ CD27^+^ B cells CD80 | 217,0 - 1,0 | 157,0 - 0,0 | 477,0 - 0,0 |
| CD20^+^ CD27^+^ B cells CTLA-4 | 915,0 - 16,0 | 985,0 - 0,0 | 506,0 - 0,0 |
| CD20^+^ CD27^+^ B cells HVEM | 104,0 - 4,0 | 75,0 - 0,0 | 266,0 - 0,0 |
| CD20^+^ CD27^+^ B cells  PD-L1 | 538,0 - 2,0 | 358,0 - 0,0 | 403,0 - 0,0 |
| CD20^-^ CD27^+^ B cells | 522,0 - 0,0 | 286,0 - 0,0 | 736,0 - 0,0 |
| CD20^-^ CD27^+^ B cells BTLA | 310,0 - 0,0 | 230,0 - 0,0 | 329,0 - 0,0 |
| CD20^-^ CD27^+^ B cells CD40 | 56,0 - 0,0 | 61,0 - 0,0 | 97,0 - 0,0 |
| CD20^-^ CD27^+^ B cells CD70 | 60,0 - 0,0 | 48,0 - 0,0 | 62,0 - 0,0 |
| CD20^-^ CD27^+^ B cells CD80 | 17,0 - 0,0 | 20,0 - 0,0 | 15,0 - 0,0 |
| CD20^-^ CD27^+^ B cells CTLA-4 | 112,0 - 0,0 | 31,0 - 0,0 | 51,0 - 0,0 |
| CD20^-^ CD27^+^ B cells HVEM | 8,0 - 0,0 | 84,0 - 0,0 | 16,0 - 0,0 |
| CD20^-^ CD27^+^ B cells  PD-L1 | 37,0 - 0,0 | 9,0 - 0,0 | 24,0 - 0,0 |
| CD4^+^ T cells | 190454,0 - 350,0 | 200687,0 - 2187,0 | 152383,0 - 18,0 |
| CD4^+^ Th cells | 185191,0 - 336,0 | 196257,0 - 2098,0 | 147637,0 - 18,0 |
| Effector Th | 6133,0 - 46,0 | 7136,0 - 83,0 | 17401,0 - 3,0 |
| Effector Th  CD28 | 5738,0 - 46,0 | 5909,0 - 10,0 | 8054,0 - 2,0 |
| Effector Th  CTLA-4 | 38,0 - 0,0 | 85,0 - 0,0 | 195,0 - 0,0 |
| Effector Th  HVEM | 39,0 - 0,0 | 52,0 - 0,0 | 98,0 - 0,0 |
| Effector Th  OX40 | 36,0 - 0,0 | 56,0 - 0,0 | 146,0 - 0,0 |
| Effector Th  PD-1 | 2415,0 - 0,0 | 1970,0 - 1,0 | 7103,0 - 0,0 |
| Effector Th  PD-L1 | 51,0 - 1,0 | 141,0 - 0,0 | 337,0 - 0,0 |
| Memory Th | 55190,0 - 143,0 | 59727,0 - 642,0 | 65990,0 - 10,0 |
| Memory Th  CD28 | 55078,0 - 143,0 | 59724,0 - 107,0 | 65806,0 - 10,0 |
| Memory Th  CTLA-44 | 188,0 - 1,0 | 433,0 - 0,0 | 460,0 - 0,0 |
| Memory Th  HVEM | 206,0 - 1,0 | 183,0 - 0,0 | 127,0 - 1,0 |
| Memory Th  OX40 | 123,0 - 0,0 | 568,0 - 0,0 | 214,0 - 0,0 |
| Memory Th  PD-1 | 8503,0 - 6,0 | 3945,0 - 22,0 | 17513,0 - 1,0 |
| Memory Th  PD-L1 | 326,0 - 2,0 | 547,0 - 3,0 | 530,0 - 0,0 |
| Naïve Th | 110692,0 - 137,0 | 87882,0 - 552,0 | 68469,0 - 4,0 |
| Naïve Th  BTLA | 107,0 - 0,0 | 103,0 - 0,0 | 137,0 - 1,0 |
| Naïve Th  CD28 | 110687,0 - 137,0 | 87871,0 - 24,0 | 68464,0 - 0,0 |
| Naïve Th  HVEM | 355,0 - 0,0 | 244,0 - 2,0 | 440,0 - 0,0 |
| Treg | 5658,0 - 14,0 | 7012,0 - 83,0 | 12696,0 - 0,0 |
| Memory Treg | 3460,0 - 10,0 | 5925,0 - 55,0 | 8852,0 - 0,0 |
| Memory Treg  CD28 | 3460,0 - 10,0 | 5893,0 - 54,0 | 8712,0 - 0,0 |
| Memory Treg  CD70 | 577,0 - 0,0 | 496,0 - 0,0 | 266,0 - 0,0 |
| Memory Treg  CD80 | 14,0 - 0,0 | 23,0 - 0,0 | 99,0 - 0,0 |
| Memory Treg  CTLA-4 | 49,0 - 0,0 | 262,0 - 0,0 | 279,0 - 0,0 |
| Memory Treg  HVEM | 18,0 - 0,0 | 51,0 - 0,0 | 106,0 - 0,0 |
| Memory Treg  OX40 | 14,0 - 0,0 | 150,0 - 0,0 | 24,0 - 0,0 |
| Memory Treg  PD-1 | 309,0 - 0,0 | 227,0 - 0,0 | 389,0 - 0,0 |
| Memory Treg  PD-L1 | 10,0 - 0,0 | 64,0 - 1,0 | 243,0 - 0,0 |
| Naïve Treg | 2228,0 - 1,0 | 3042,0 - 11,0 | 2694,0 - 0,0 |
| Naïve Treg  BTLA | 19,0 - 0,0 | 40,0 - 0,0 | 137,0 - 0,0 |
| Naïve Treg  CD28 | 2228,0 - 1,0 | 3042,0 - 10,0 | 2691,0 - 0,0 |
| Naïve Treg  CTLA-4 | 60,0 - 0,0 | 565,0 - 0,0 | 2248,0 - 0,0 |
| Naïve Treg  HVEM | 62,0 - 0,0 | 111,0 - 0,0 | 137,0 - 0,0 |
| Naïve Treg  PD-L1 | 41,0 - 0,0 | 74,0 - 0,0 | 125,0 - 0,0 |
| CD8^+^ T cells | 64653,0 - 117,0 | 77170,0 - 792,0 | 62670,0 - 37,0 |
| Effector CD8^+^ T cells | 3267,0 - 5,0 | 7244,0 - 14,0 | 15094,0 - 8,0 |
| Effector CD8^+^ T cells  CD28 | 682,0 - 2,0 | 5130,0 - 0,0 | 1070,0 - 0,0 |
| Effector CD8^+^ T cells  HVEM | 30,0 - 0,0 | 45,0 - 0,0 | 61,0 - 0,0 |
| Effector CD8^+^ T cells  PD-1 | 527,0 - 0,0 | 1737,0 - 0,0 | 1034,0 - 0,0 |
| Effector CD8^+^ T cells  PD-L1 | 98,0 - 0,0 | 44,0 - 0,0 | 130,0 - 0,0 |
| Memory CD8^+^ T cells | 10980,0 - 16,0 | 48764,0 - 174,0 | 18395,0 - 5,0 |
| Memory CD8^+^ T cells  CD28 | 9076,0 - 8,0 | 6808,0 - 9,0 | 9386,0 - 0,0 |
| Memory CD8^+^ T cells  HVEM | 45,0 - 0,0 | 132,0 - 0,0 | 165,0 - 0,0 |
| Memory CD8^+^ T cells  PD-1 | 3590,0 - 1,0 | 3519,0 - 0,0 | 2304,0 - 0,0 |
| Memory CD8^+^ T cells  PD-L1 | 138,0 - 0,0 | 125,0 - 1,0 | 256,0 - 0,0 |
| Naïve CD8^+^ T cells | 30154,0 - 75,0 | 24353,0 - 233,0 | 29774,0 - 6,0 |
| Naïve CD8^+^ T cells  BTLA | 78,0 - 0,0 | 87,0 - 1,0 | 89,0 - 1,0 |
| Naïve CD8^+^ T cells  CD28 | 266,0 - 1,0 | 591,0 - 1,0 | 231,0 - 0,0 |
| Naïve CD8^+^ T cells  HVEM | 130,0 - 0,0 | 333,0 - 1,0 | 427,0 - 0,0 |
| Terminal effector CD8^+^ T cells | 9921,0 - 6,0 | 21018,0 - 9,0 | 26110,0 - 11,0 |
| Terminal effector CD8^+^ T cells  HVEM | 17,0 - 0,0 | 130,0 - 0,0 | 114,0 - 0,0 |
| Terminal effector CD8^+^ T cells  PD-1 | 454,0 - 0,0 | 548,0 - 0,0 | 398,0 - 0,0 |
| Terminal effector CD8^+^ T cells  PD-L1 | 215,0 - 0,0 | 298,0 - 0,0 | 118,0 - 0,0 |
| InfDCS | 2678,0 - 7,0 | 1223,0 - 3,0 | 2414,0 - 3,0 |
| InfDCS  BTLA | 32,0 - 0,0 | 32,0 - 0,0 | 19,0 - 0,0 |
| InfDCS  CD40 | 12,0 - 0,0 | 17,0 - 0,0 | 25,0 - 0,0 |
| InfDCS  HVEM | 5,0 - 0,0 | 7,0 - 0,0 | 6,0 - 0,0 |
| Classical monocytes | 26894,0 - 43,0 | 22645,0 - 20,0 | 24711,0 - 190,0 |
| Classical monocytes  CD40 | 80,0 - 0,0 | 143,0 - 0,0 | 75,0 - 1,0 |
| Classical monocytesHVEM | 85,0 - 0,0 | 188,0 - 0,0 | 153,0 - 0,0 |
| Intermediate monocytes | 1666,0 - 102,0 | 6577,0 - 40,0 | 16751,0 - 17,0 |
| Intermediate monocytes  CD40 | 5,0 - 0,0 | 79,0 - 0,0 | 37,0 - 0,0 |
| Intermediate monocytes  HVEM | 13,0 - 0,0 | 184,0 - 0,0 | 95,0 - 0,0 |
| Non-classical monocytes | 2704,0 - 39,0 | 2609,0 - 71,0 | 4048,0 - 42,0 |
| Non-classical monocytes  CD40 | 17,0 - 0,0 | 80,0 - 0,0 | 46,0 - 0,0 |
| Non-classical monocytes  HVEM | 24,0 - 0,0 | 70,0 - 0,0 | 323,0 - 0,0 |
| NK^bright^ | 2896,0 - 102,0 | 5856,0 - 88,0 | 3040,0 - 47,0 |
| NK^bright^  BTLA | 57,0 - 0,0 | 118,0 - 0,0 | 159,0 - 0,0 |
| NK^bright^  CD70 | 89,0 - 0,0 | 45,0 - 0,0 | 66,0 - 0,0 |
| NK^bright^  HVEM | 110,0 - 0,0 | 40,0 - 1,0 | 83,0 - 0,0 |
| NK^bright^  PD-1 | 53,0 - 0,0 | 70,0 - 0,0 | 97,0 - 1,0 |
| NK^bright^  PD-L1 | 15,0 - 0,0 | 46,0 - 0,0 | 62,0 - 1,0 |
| NK^dim^ | 44980,0 - 437,0 | 86936,0 - 86,0 | 31284,0 - 230,0 |
| NK^dim^  BTLA | 197,0 - 4,0 | 192,0 - 0,0 | 207,0 - 4,0 |
| NK^dim^  CD70 | 364,0 - 2,0 | 1334,0 - 1,0 | 427,0 - 3,0 |
| NK^dim^  HVEM | 374,0 - 0,0 | 243,0 - 4,0 | 237,0 - 5,0 |
| NK^dim^  PD-1 | 388,0 - 1,0 | 566,0 - 2,0 | 397,0 - 5,0 |
| NK^dim^  PD-L1 | 473,0 - 1,0 | 417,0 - 5,0 | 810,0 - 6,0 |
| CD56^+^ CD3^+^ cells | 58339,0 - 22,0 | 16741,0 - 20,0 | 24325,0 - 45,0 |

**Supplementary Table S4. Cell population proportions and their IC expression differ in MS patients and HCs.**

|  | **Healthy Donors** | **RRMS** |  |
| --- | --- | --- | --- |
| **Cell populations** | | | |
|  | **Mean (%) ± SD** | **Mean (%) ± SD** | ***p-value*** |
| T helper CD4+ | 95,11±1,61 | 93,48±1,87 | 0,002 |
| Memory T helper | 37,93±8,75 | 30,77±8,92 | 0,008 |
| T reg | 4,82±1,57 | 6,52±1,87 | 0,002 |
| Memory CD8^+^ cells | 39,08 ± 20,29 | 14,23 ± 8,00 | <0,001 |
| Naïve CD8^+^ cells | 11,34 ± 9,13 | 43,25 ± 19,84 | <0,001 |
| InfDCs | 7,06 ± 2,79 | 4,59 ± 2,65 | <0,001 |
| **ICs expression** | | | |
|  | **Mean (%) ± SD** | **Mean (%) ± SD** | ***p-value*** |
| **CD20^+^CD27^-^ B cells** |  |  |  |
| CD40 | 97,96 ± 1,85 | 94,40 ± 14,09 | <0,001 |
| HVEM | 1,55 ± 1,03 | 0,80 ± 0,57 | 0,002 |
| PD-1 | 1,51 ± 0,82 | 2,72 ± 1,82 | 0,005 |
| **CD20^+^CD27^+^ B cells** |  |  |  |
| CD80 | 2,36 ± 0,96 | 3,80 ± 2,01 | 0,005 |
| **CD20^-^CD27^+^ B cells** |  |  |  |
| BTLA | 22,98 ± 21,13 | 50,30 ± 25,07 | <0,001 |
| CD40 | 11,96 ± 6,97 | 29,20 ± 20,91 | <0,001 |
| CD70 | 27,85 ± 21,55 | 10,47 ± 8,50 | <0,001 |
| CTLA-4 | 14,47 ± 8,08 | 5,42 ± 8,60 | <0,001 |
| PD-L1 | 7,96 ± 7,38 | 3,86 ± 5,10 | 0,011 |
| **Effector T helper** |  |  |  |
| OX40 | 0,30 ± 0,21 | 0,85 ± 0,91 | 0,017 |
| PD-1 | 14,27 ± 13,06 | 6,71 ± 11,23 | 0,016 |
| PD-L1 | 0,62 ± 0,48 | 1,33 ± 1,05 | 0,003 |
| **Memory T helper** |  |  |  |
| CTLA-4 | 0,40 ± 0,30 | 0,29 ± 0,33 | 0,049 |
| PD-1 | 15,69 ± 6,61 | 6,44 ± 3,95 | <0,001 |
| **Naïve T helper** |  |  |  |
| BTLA | 0,21 ± 0,49 | 0,31 ± 0,41 | 0,007 |
| HVEM | 0,49 ± 0,69 | 0,59 ± 0,46 | 0,021 |
| **Memory Treg** |  |  |  |
| CD70 | 12,67 ± 5,89 | 5,17 ± 4,10 | <0,001 |
| CD80 | 0,41 ± 0,41 | 0,95 ± 1,07 | 0,016 |
| HVEM | 0,39 ± 0,45 | 1,04 ± 1,46 | 0,003 |
| OX40 | 0,26 ± 0,28 | 0,69 ± 0,61 | 0,002 |
| PD-L1 | 0,48 ± 0,66 | 1,30 ± 1,23 | 0,002 |
| **Naïve Treg** |  |  |  |
| BTLA | 1,35 ± 2,51 | 3,38 ± 2,67 | 0,002 |
| CD28 | 99,99 ± 0,02 | 99,42 ± 1,28 | 0,002 |
| **Terminal Effector CD8^+^** |  |  |  |
| HVEM | 0,50 ± 1,13 | 1,31 ± 2,41 | <0,001 |
| PD-1 | 15,36 ± 15,46 | 2,90 ± 2,93 | 0,001 |
| **Memory CD8^+^** |  |  |  |
| CD28 | 0,57 ± 0,88 | 80,04 ± 16,58 | <0,001 |
| **Naïve CD8^+^** |  |  |  |
| BTLA | 3,31 ± 4,12 | 0,85 ± 1,15 | <0,001 |
| CD28 | 2,02 ± 7,58 | 1,39 ± 1,57 | <0,001 |
| HVEM | 2,82 ± 3,62 | 0,84 ± 0,77 | 0,011 |
| **InfDCs** |  |  |  |
| BTLA | 1,45 ± 1,38 | 6,20 ± 5,31 | <0,001 |
| CD40 | 0,77 ± 2,26 | 2,08 ± 2,19 | <0,001 |
| HVEM | 0,32 ± 0,48 | 0,81 ± 1,22 | 0,038 |
| **Classical Monocytes** |  |  |  |
| CD40 | 0,43 ± 0,34 | 1,07 ± 1,02 | 0,003 |
| HVEM | 0,28 ± 0,25 | 0,57 ± 0,51 | 0,006 |
| **Intermediate Monocytes** |  |  |  |
| CD40 | 0,39 ± 0,76 | 1,89 ± 2,60 | <0,001 |
| HVEM | 0,47 ± 0,59 | 1,94 ± 8,48 | 0,002 |
| **Non Classical Monocytes** |  |  |  |
| CD40 | 1,37 ± 4,03 | 2,08 ± 7,80 | 0,002 |
| **Natural Killer^bright^** |  |  |  |
| CD70 | 3,20 ± 1,63 | 1,12 ± 1,21 | <0,001 |
| PD-1 | 1,15 ± 1,00 | 2,56 ± 1,71 | <0,001 |
| PD-L1 | 0,58 ± 0,45 | 2,93 ± 3,55 | <0,001 |
| **Natural Killer^dim^** |  |  |  |
| CD70 | 1,16 ± 0,67 | 0,82 ± 0,82 | 0,029 |
|  |  |  |  |

**Supplementary Table S5. Immune checkpoints expression across treatments.**

| **Mean (%) ± SD** | ***p-value*** | | | | | | | **Mean (%) ± SD** |
| --- | --- | --- | --- | --- | --- | --- | --- | --- |
| **B cells** |  | **Natalizumab** | **Teriflunomide** | **DMF** | **Anti-CD20** | **Cladribine** |  |  |
| 3,68 ± 11,63 | **Natalizumab** |  |  |  |  |  | **Natalizumab** |  |
| -24,22 ± 16,87 | **Teriflunomide** | 0,005 |  |  |  |  | **Teriflunomide** |  |
| 3,36 ± 12,35 | **DMF** |  | 0,007 |  |  |  | **DMF** |  |
| -60,18 ± 28,61 | **Anti-CD20** | <0,001 |  | <0,001 |  |  | **Anti-CD20** |  |
| -16,68 ± 14,53 | **Cladribine** | 0,004 |  | 0,007 | 0,013 |  | **Cladribine** |  |
| **CD20^+^CD27^-^ B cells** |  | **Natalizumab** | **Teriflunomide** | **DMF** | **Anti-CD20** | **Cladribine** |  | **CD40** |
|  | **Natalizumab** |  |  |  |  |  | **Natalizumab** | -5,26 ± 7,48 |
|  | **Teriflunomide** |  |  |  |  |  | **Teriflunomide** | -20,48 ± 39,26 |
|  | **DMF** |  |  |  | 0,012 |  | **DMF** | -1,99 ± 6,81 |
|  | **Anti-CD20** |  |  |  |  |  | **Anti-CD20** | -60,47 ± 4 8,43 |
|  | **Cladribine** |  |  |  |  |  | **Cladribine** | -5,25 ± 7,74 |
|  |  | **Natalizumab** | **Teriflunomide** | **DMF** | **Anti-CD20** | **Cladribine** |  | **HVEM** |
|  | **Natalizumab** |  |  |  |  |  | **Natalizumab** | -0,01 ± 0,66 |
|  | **Teriflunomide** |  |  |  | 0,018 |  | **Teriflunomide** | 0,45 ± 0,84 |
|  | **DMF** |  |  |  |  |  | **DMF** | -0,15 ± 0,82 |
|  | **Anti-CD20** |  |  |  |  | 0,009 | **Anti-CD20** | -0,78 ± 0,68 |
|  | **Cladribine** |  |  |  |  |  | **Cladribine** | 0,25 ± 0,97 |
|  |  | **Natalizumab** | **Teriflunomide** | **DMF** | **Anti-CD20** | **Cladribine** |  | **PD-1** |
|  | **Natalizumab** |  |  |  | 0,012 |  | **Natalizumab** | 6,82 ± 24,43 |
|  | **Teriflunomide** |  |  | 0,037 | <0,001 |  | **Teriflunomide** | 45,84 ± 50,05 |
|  | **DMF** |  |  |  | 0,037 | 0,037 | **DMF** | -0,39 ± 1,58 |
|  | **Anti-CD20** |  |  |  |  | <0,001 | **Anti-CD20** | -2,66 ± 1,21 |
|  | **Cladribine** |  |  |  |  |  | **Cladribine** | 27,92 ± 40,80 |
| **CD20^+^CD27^+^ B cells** |  | **Natalizumab** | **Teriflunomide** | **DMF** | **Anti-CD20** | **Cladribine** |  | **BTLA** |
| 28,49 ± 27,86 | **Natalizumab** |  |  |  |  |  | **Natalizumab** | 1,56 ± 32,18 |
| 22,75 ± 30,67 | **Teriflunomide** |  |  |  |  |  | **Teriflunomide** | 20,22 ± 35,02 |
| -2,90 ± 10,98 | **DMF** | 0,018 |  |  |  | 0,016 | **DMF** | -18,45 ± 34,18 |
| -11,67 ± 35,51 | **Anti-CD20** | 0,002 | 0,027 |  |  |  | **Anti-CD20** | 5,79 ± 53,86 |
| -9,20 ± 21,69 | **Cladribine** | <0,001 | 0,018 |  |  |  | **Cladribine** | 26,77 ± 43,18 |
| **CD20^-^CD27^+^ B cells** |  | **Natalizumab** | **Teriflunomide** | **DMF** | **Anti-CD20** | **Cladribine** |  |  |
| 0,34 ± 0,77 | **Natalizumab** |  |  |  |  |  | **Natalizumab** |  |
| 1,69 ± 2,20 | **Teriflunomide** |  |  |  |  |  | **Teriflunomide** |  |
| -0,03 ± 1,04 | **DMF** |  |  |  |  |  | **DMF** |  |
| 49,65 ± 34,85 | **Anti-CD20** | 0,004 |  | 0,001 |  |  | **Anti-CD20** |  |
| 0,00 ± 2,01 | **Cladribine** |  |  |  | <0,001 |  | **Cladribine** |  |
| **Memory T helper** |  | **Natalizumab** | **Teriflunomide** | **DMF** | **Anti-CD20** | **Cladribine** |  | **HVEM** |
|  | **Natalizumab** |  |  |  |  |  | **Natalizumab** | 0,31 ± 0,67 |
|  | **Teriflunomide** |  |  | 0,037 |  |  | **Teriflunomide** | 0,58 ± 0,70 |
|  | **DMF** |  |  |  |  | 0,016 | **DMF** | -0,39 ± 0,58 |
|  | **Anti-CD20** |  |  |  |  |  | **Anti-CD20** | 0,28 ± 0,96 |
|  | **Cladribine** |  |  |  |  |  | **Cladribine** | 0,98 ± 2,41 |
| **Effector T helper** |  | **Natalizumab** | **Teriflunomide** | **DMF** | **Anti-CD20** | **Cladribine** |  | **CTLA-4** |
|  | **Natalizumab** |  |  | 0,039 |  |  | **Natalizumab** | 0,15 ± 0,62 |
|  | **Teriflunomide** |  |  | 0,039 |  |  | **Teriflunomide** | 0,00 ± 0,24 |
|  | **DMF** |  |  |  |  | 0,007 | **DMF** | 0,98 ± 0,78 |
|  | **Anti-CD20** |  |  |  |  |  | **Anti-CD20** | 0,48 ± 0,55 |
|  | **Cladribine** |  |  |  |  |  | **Cladribine** | 0,10 ± 1,18 |
| **Memory CD8^+^** |  | **Natalizumab** | **Teriflunomide** | **DMF** | **Anti-CD20** | **Cladribine** |  | **PD-1** |
|  | **Natalizumab** |  |  | 0,030 | 0,023 |  | **Natalizumab** | -2,14 ± 8,94 |
|  | **Teriflunomide** |  |  | 0,023 | 0,023 |  | **Teriflunomide** | -3,84 ± 5,72 |
|  | **DMF** |  |  |  |  |  | **DMF** | 5,62 ± 8,09 |
|  | **Anti-CD20** |  |  |  |  | 0,041 | **Anti-CD20** | 8,45 ± 10,08 |
|  | **Cladribine** |  |  |  |  |  | **Cladribine** | 0,41 ± 6,38 |
| **Natural Killer^dim^** |  | **Natalizumab** | **Teriflunomide** | **DMF** | **Anti-CD20** | **Cladribine** |  | **PD-L1** |
|  | **Natalizumab** |  |  |  |  |  | **Natalizumab** | 1,48 ± 2,09 |
|  | **Teriflunomide** |  |  | 0,031 | 0,046 |  | **Teriflunomide** | 2,51 ± 2,43 |
|  | **DMF** |  |  |  |  |  | **DMF** | -0,25 ± 2,48 |
|  | **Anti-CD20** |  |  |  |  |  | **Anti-CD20** | 0,74 ± 2,72 |
|  | **Cladribine** |  |  |  |  |  | **Cladribine** | 1,07 ± 1,30 |
|  |  |  |  |  |  |  |  |  |

*Each box corresponds to a cell type (left column) and the immune checkpoints (ICs) expressed by that cell type (right column). The boxes also include the p-values for significant differences between treatments based on parametric or non-parametric tests. The first and last columns show the mean (%) ± SD of the percentages of the cell types and their corresponding ICs expression (labeled above). In the central matrix, the bottom section displays the p-values for the cell populations, while the top section shows the ICs.

**Supplementary material S6 Linear regression data:**

**Association with disability progression**

BTLA expression in CD20^-^CD27^+^ B cells (estimate = 35.68, 95% CI [8.93, 62.43], p = 0.0108).

CTLA-4 expression in CD20^-^CD27^+^ B cells (estimate = -10.93, 95% CI [-20.13, -1.72], p = 0.0217).

Frequency of Th cells (estimate = -2.07, 95% CI [-3.91, -0.24], p = 0.0281).

HVEM expression in Th cells (estimate = 0.59, 95% CI [0.03, 1.14], p = 0.0403).

CTLA-4 in memory Th cells (estimate = -0.32, 95% CI [-0.59, -0.05], p = 0.0227).

Treg frequency (estimate = 2.13, 95% CI [0.33, 3.92], p = 0.0222).

CTLA-4 expression in memory Tregs (estimate = -2.28, 95% CI [-4.38, -0.18], p = 0.0343).

PD-L1 in memory Tregs (estimate = 1.15, 95% CI [0.19, 2.11], p = 0.0210).

CD40 expression in non-classical monocytes (estimate = 0.87, 95% CI [0.22, 1.52], p = 0.0107).

**Association with new MRI lesions**

Soluble plasma PD-1 expression (estimate = 32.65, 95% CI [11.41, 53.89], p = 0.0038).

**Association with relapses**

Soluble plasma IL-10 levels (estimate = -47.54, 95% CI [-94.27, -0.80], p = 0.0466).

BTLA in CD20^+^CD27^-^ B cells (estimate = 35.91, 95% CI [3.19, 68.62], p = 0.0326).

BTLA in CD20^+^CD27^+^ B cells (estimate = 31.87, 95% CI [0.34, 63.39], p = 0.0477).

BTLA expression in naïve Treg cells (estimate = -3.36, 95% CI [-6.03, -0.69], p = 0.0155).

**Supplementary Table S7. Immune cell subset changes by therapy response**
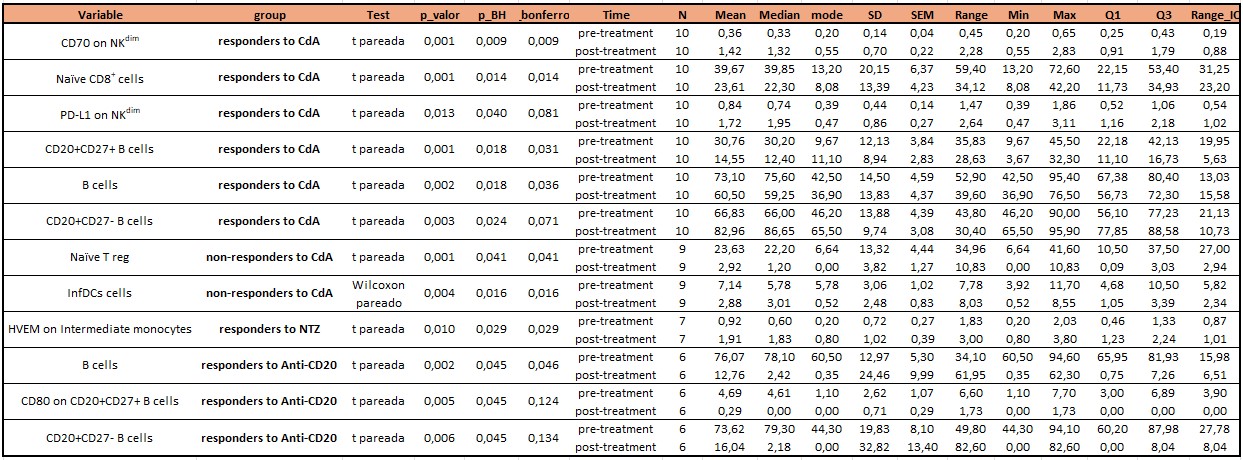

Supplement: Supplementary file 1 [file DataSheet1.docx]
